# Farmer perceptions of the vulnerabilities of traditional livestock farming systems under global change

**DOI:** 10.1007/s13280-025-02150-8

**Published:** 2025-02-26

**Authors:** Zebensui Morales‐Reyes, Jomar M. Barbosa, José A. Sánchez‐Zapata, Irene Pérez-Ibarra

**Affiliations:** 1 Departamento de Biología Animal, Edafología y Geología, Facultad de Ciencias, Universidad de La Laguna (ULL), Avenida Astrofísico Francisco Sánchez, s/n, 38206 San Cristóbal de La Laguna (Tenerife), Canary Islands Spain; 2 Instituto de Estudios Sociales Avanzados (IESA), CSIC, Campo Santo de los Mártires, 7, 14004 Córdoba, Spain; 3 Department of Applied Biology, Miguel Hernández University of Elche, Avda. Universidad s/n, 03202 Elche, Spain; 4 Centro de Investigación e Innovación Agroalimentaria y Agroambiental (CIAGRO-UMH), Miguel Hernández University of Elche, Ctra. de Beniel km 3.2, 03312 Orihuela, Spain; 5 Departamento de Ciencias Agrarias y del Medio Natural, Universidad de Zaragoza, Calle Miguel Servet, 177, 50013 Zaragoza, Spain; 6 Grupo de Sistemas Socioecológicos Agrarios, Instituto Agroalimentario de Aragón, Calle Miguel Servet, 177, 50013 Zaragoza, Spain

**Keywords:** Agroecosystems, Climate change, Coupled Infrastructure Systems framework, Farming intensification, Pastoralism, Rewilding

## Abstract

The continuity of traditional extensive livestock farming is being challenged by rapid socioeconomic and environmental changes, threatening livelihoods and ecosystem services critical to food security and sustainability. We conducted a large-scale assessment involving 255 livestock farmers across six extensive livestock farming systems in Spain to understand their perceptions of vulnerabilities. Using the Coupled Infrastructure Systems framework, we identified 24 different vulnerabilities, mainly caused by external socioeconomic and biophysical disturbances, such as resource costs, low profitability of livestock products, climate variability, and conflicts with wildlife. The main factors explaining these vulnerabilities were primary productivity, farm location, presence of large predators, and climatic conditions. The findings highlight the complex interplay of these factors and provide important insights for the maintenance of extensive livestock farming systems in Europe. This information is crucial for informing policy decisions aimed at supporting these farming systems and ensuring their contribution to food security, sustainability and biodiversity conservation.

**Supplementary Information:**

The online version contains supplementary material available at 10.1007/s13280-025-02150-8.

## Introduction

Extensive livestock farming systems, which use local natural resources through grazing and include traditional practices such as transhumance, provide essential ecosystem services (Rodríguez-Ortega et al. 2014; Dumont et al. 2019) and support the livelihoods of millions of rural families worldwide (FAO 2009). These systems contribute to several Sustainable Development Goals (SDGs) (United Nations 2016), including no poverty (SDG 1), zero hunger (SDG 2), decent work and economic growth (SDG 8), climate action (SDG 13), and life on land (SDG 15). These extensive livestock systems also enhance soil quality and sequester carbon by maintaining healthy grasslands, which also supports biodiversity by preserving open landscapes, aiding in fire prevention and reducing habitat degradation (e.g., Rouet-Leduc et al. 2021; Su et al. 2023). Their adaptability to natural ecosystem functioning makes them resilient to climate change, providing a sustainable alternative to more intensive agricultural systems (e.g., Bonilla-Cedrez et al. 2023; Tugjamba et al. 2023). This combination of ecological benefits and adaptability underscores their role in addressing global environmental challenges.

In recent years, there has been growing interest in studying the vulnerability of these extensive livestock systems because climate change, globalization, and multiple social, environmental, economic, institutional and political changes threaten their contribution to food security, human well-being, and environmental conservation (López-i-Gelats et al. 2016; Alary et al. 2022). In this context, there is an urgent need for studies that contribute to the current scientific and public debate on how farming intensification changes traditional practices (Clay et al. 2020), how climate change impacts extensive livestock production (e.g., Rojas-Downing et al. 2017) and how rewilding creates human-wildlife conflicts (e.g., Wolf and Ripple 2018). These global pressures pose significant threats to the long-term sustainability of these systems by altering both their environmental and socioeconomic contexts. Here, we examine the vulnerability of extensive livestock farming systems in face of these different social and ecological challenges, recognizing the potential existence of regional and local differences (e.g., Thomasz et al. 2020; Plieninger et al. 2021).

Vulnerability refers to the degree to which social-ecological systems are likely to be harmed due to disturbances, such as drought, high production costs, or low profitability (Urruty et al. 2016). Some traditional farming systems have persisted over long periods because of their adaptability to certain natural and social external disturbances (Janssen et al. 2007). For example, transhumance systems (an ancient pastoralist practice) have long adapted to the natural environmental variability by following regular routes coupled with seasonal changes in pasture availability (Manzano and Casas 2020). However, climate change, rural–urban outmigration, intensification of production systems, and changes in the profitability of livestock products have brought new challenges and vulnerabilities to these traditional livestock farming systems (e.g., Oteros-Rozas et al. 2019).

Growth trends in the livestock sector are highly dynamic and heterogeneous worldwide. In some parts of the world, such as Africa, Latin America, and tropical and subtropical Asia, the livestock sector is growing rapidly. In other places, particularly in Europe and North America, the livestock sector is stable or even declining (FAO 2017). Particularly in Spain, Portugal and Italy (Mediterranean region), extensive livestock farming systems have declined sharply in recent decades (Hostiou et al. 2020), while the intensification of livestock production systems is increasing (e.g., Bernués et al. 2011; Fig. S1). To better understand the complex social-ecological interactions shaping traditional extensive livestock farming systems, there is an increasing need for integrated and participatory approaches that involve actors in disentangling the relative importance of social, ecological and economic factors in ensuring the sustainability of these systems (Mielke et al. 2016; Manzano et al. 2021).

**Fig. 1:**
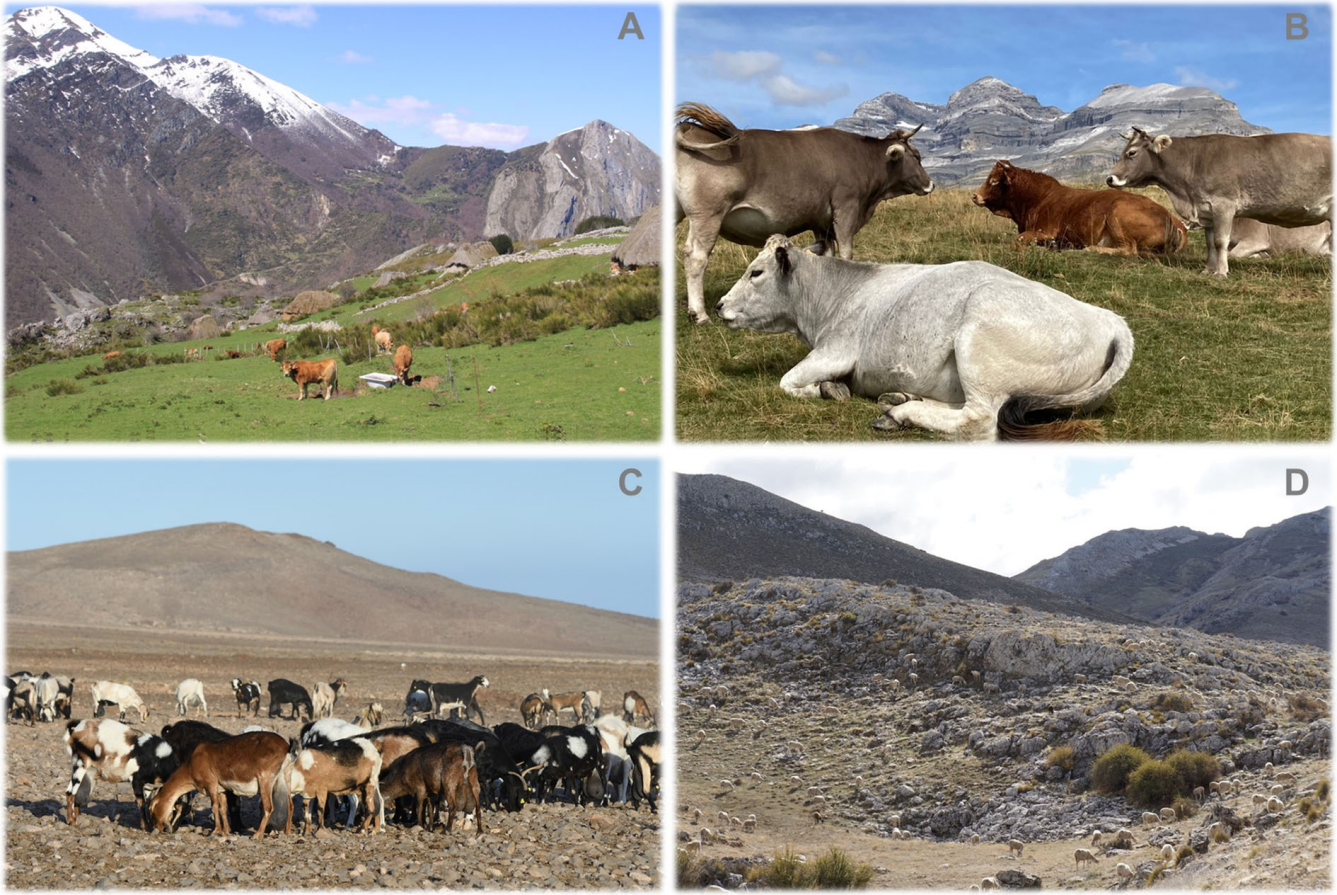
Photographs from different study areas: several cattle in the Cantabrian Mountains (**A**) and the Pyrenees (**B**), a herd of goats in Fuerteventura on the Canary Islands and a herd of sheep in the Sierras de Cazorla, Segura y Las Villas Natural Park (**D**). *Credits*: Zebensui Morales (**A**), José A. Sánchez (**B**), Manuel de la Riva (**C**) and Eduardo García (**D**)

**Fig. 2:**
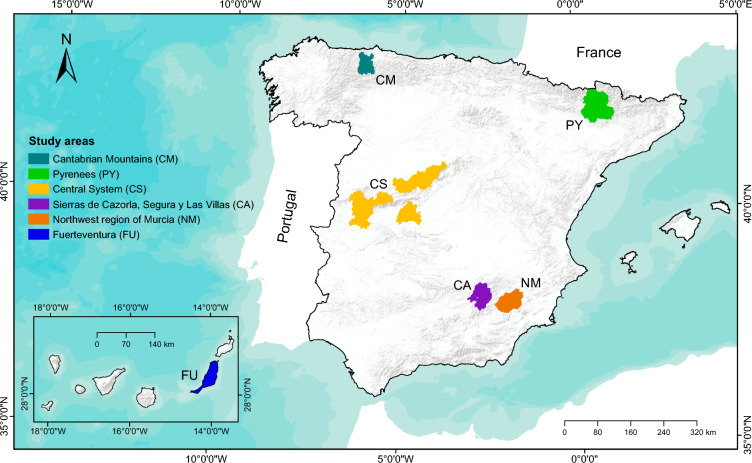
Location of the six study areas in Spain

In this study, the objectives are: (1) to assess livestock farmers’ perceptions of the main vulnerabilities of traditional extensive livestock farming systems in Spain (Fig. 1), and (2) to analyze the social and ecological factors responsible for such vulnerabilities. These objectives are essential for finding robust and innovative solutions that enhance the sustainability of these systems. Specifically, we use the Coupled Infrastructure Systems (CIS) framework (Anderies et al. 2016) to systematically examine these vulnerabilities as perceived by farmers. The CIS framework, based on systems thinking (Walker and Salt 2012), provides a structured approach to connecting natural resource use, including common-pool resources (Ostrom 1990), with resilience theory in complex social-ecological systems (Janssen and Anderies 2023). Resilience theory, originating from Holling (1973), refers to a system's ability to absorb disturbances and reorganize while undergoing change. This approach helps to analyze how different infrastructures (natural, human-made) interact, and how these interactions affect system vulnerabilities to both internal and external disturbances (Anderies et al. 2016; Janssen and Anderies 2023). This framework effectively organizes, categorizes, and interprets the dynamics of social-ecological systems and their links, as well as the effects of internal and external sources of disturbances on system sustainability (see details in the Materials and Methods section). By highlighting farmers' perspectives on vulnerabilities, this study advances academic knowledge and contributes to the development of integrated and participatory research approaches. These approaches, in turn, can inform evidence-based policymaking aimed at enhancing the resilience and sustainability of livestock farming systems. These insights can support policymakers to develop policies for enhancing the resilience and sustainability of livestock farming systems. We first use the CIS framework to identify vulnerabilities. Then, we use the redundancy analyses to identify the main social and ecological factors driving the vulnerabilities perceived by farmers. And finally, we discuss regional level differences in the perceived vulnerabilities of traditional extensive livestock farming located at a wide range of environmental conditions, cultural heritage, and productive context.

## Materials and methods

We surveyed livestock farmers (hereafter, farmers) from traditional extensive livestock systems (Fig. 1) in six mountainous areas in Spanish (Fig. 2): The Cantabrian Mountains, the Pyrenees, the Central System, Sierras de Cazorla, Segura y Las Villas Natural Park (hereafter, Cazorla), the northwest region of Murcia (hereafter, Murcia) on peninsular Spain, and Fuerteventura in the Canary Islands (Fig. 1). Here, traditional extensive livestock systems refer to those that utilize natural resources with relatively low external inputs, primarily through grazing, and are characterized by species and breeds adapted to the local environment, as well as diverse pastures based on spatial and temporal availability. The term "traditional" indicates practices deeply rooted in local history and culture, reflecting long-established methods of farming in these regions. In total, we interviewed 255 livestock farmers between 2012 and 2016, ensuring representation of the total population of farmers in each study area (Appendix S1, Table S1). The questionnaire was pre-tested on a small sample of farmers in the northwest region of Murcia to improve its readability and clarity (see Appendix S2).

The data collection was systematically divided into three main stages. First, we randomly selected farmers of extensive livestock farming systems from the Spanish General Register of Livestock Farms. Second, we obtained farmers’ contact information from the local sanitary authorities. Third, we conducted face-to-face surveys with farmers in their farms or in the surrounding pasture area. On the few occasions when access to farmers was limited, we used snowball sampling to ensure broader coverage (see Appendix S2 for details on the sampling strategy).

All farmers were informed that their participation was voluntary and anonymous. The study was conducted in accordance with the ethical principles outlined in the Belmont Report (National Commission for the Protection of Human Subjects of Biomedical and Behavioral Research 1979). We asked the open-ended question, “*In your opinion, what are the main problems you face on your farm?*” to capture the vulnerabilities of the livestock farming systems as they were perceived by the surveyed farmers in our study areas (original question: *“En su opinión, ¿cuáles son los principales problemas a los que se enfrenta con su explotación?”*). In asking this question, we explained to the surveyed farmers the scope of the question, and the farmers were encouraged to discuss vulnerabilities in the present, but also in relation to the future. The farmers’ responses were recorded in handwritten form during the interviews, which lasted an average of 45 min and were conducted in Spanish. Here, vulnerability refers to the degree to which extensive livestock farming systems are likely to be harmed due to problems mentioned by farmers (e.g., high production costs).

### Vulnerabilities of traditional extensive livestock farming systems

**Fig. 3:**
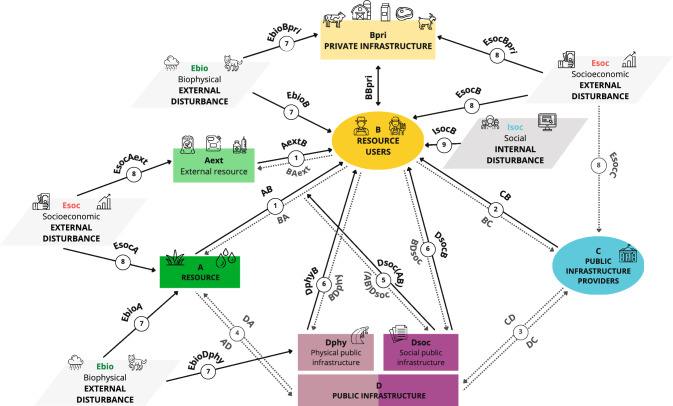
Adaptation of Coupled Infrastructure Systems (Anderies et al. 2016) to traditional livestock farming systems. Humans (B and C) are shown as circles. The squares show two forms of human-made capital (Dphy and Dsoc), resources (A and Aext) and private infrastructure (Bpri). The parallelograms represent internal disturbances (Isoc) and external disturbances (Esoc and Ebio). Black solid arrows indicate the direction of the interaction between the components involved as identified by surveyed farmers, whereas dashed arrows show interactions proposed by Anderies et al. (2004) that were not mentioned by surveyed farmers

We applied the CIS framework (Anderies et al. 2016), previously known as the social-ecological robustness framework (Anderies et al. 2004), to analyze the vulnerabilities of traditional livestock farming systems as perceived by farmers (Fig. 3; Tables 1, 2 and 3). This approach enabled us to address our first objective by identifying the system components associated with the vulnerabilities identified by the interviewees, which helps to understand the interconnected nature of problems within livestock farming systems and the dynamics of the systems studied. We coded each response as a link between different components of the CIS framework (see Tables 1, 2 and 3 for coding examples). When adapted to livestock farming systems, the CIS framework represents resources (i.e., pastures, water) and external resources (e.g., food inputs, fuel, veterinary drugs) used by resource users (i.e., farmers). Resource users have their own private infrastructure (e.g., livestock herd, livestock products) and use public infrastructure (e.g., roads). Two components are composed of humans: resource users and public infrastructure providers (e.g., government). Infrastructures can be private (e.g., livestock herd) or public including physical (e.g., roads) and social (i.e., norms and rules). There are two types of external disturbances: biophysical (e.g., climate, natural vegetation, wildlife) and socioeconomic (e.g., market fluctuations, subsidies, policy changes) that impact the components of the system. This framework also considers internal disturbances (e.g., generational renewal) and the links between all of these components.

**Table 1:** Components of the Coupled Infrastructure System involved in traditional livestock farming systems. Adapted from Anderies et al. (2004, 2016)

Component		Coding examples
A. Resource		Pasture, water
		Food inputs (i.e., cereals and supplementary feed), others external inputs (i.e., fuel, veterinary drugs)
B. Resource Users		Farmers
		Farms, livestock, livestock products (e.g., meat, milk, wool)
C. Public infrastructure providers		Government
D. Public infrastructure		Institutions (i.e., rules, regulations)
		Roads
E. External disturbances		Climate, natural vegetation, wildlife
		Credits and taxes, economic crisis, free riding (i.e., illegal workers, robbers), insurances, markets, subsidies
I. Internal disturbances		Demanding job, labor shortage, Weak generational renewal

**Table 2:** Links (i.e., vulnerabilities) of the Coupled Infrastructure System involved in traditional livestock farming systems. Coding examples of the links mentioned by surveyed farmers are shown (see Table S2 for complete list of coding examples)

Links		Coding examples
*Resource users-related vulnerabilities*		
Between resource users and private infrastructure		Dung accumulation
*Resource-related vulnerabilities*		
(1) Between resource and resource users		Pasture shortage
(1) Between external resource and resource users		Cereal shortage, low quality of animal food
*Public infrastructure-related vulnerabilities*		
(2) Between public infrastructure providers and resource users		Government´s lack of support to the sector
(5) Between public infrastructure and resource dynamics		Land management
(6) Between resource users and physical public infrastructure		Lack of roads and roads in disrepair
(6) Between resource users and social public infrastructure		Bureaucracy, restrictions to animal movements

**Table 3:** Links (i.e., vulnerabilities) of the Coupled Infrastructure System involved in traditional livestock farming systems. Coding examples of the links mentioned by surveyed farmers are shown (see Table S2 for complete list of coding examples)

Links		Coding examples
*Biophysical external disturbances*		
(7) Biophysical external disturbances on resource		Drought, wild boar rooting
(7) Biophysical external disturbances resource users		Inclement weather
(7) Biophysical external disturbances on private infrastructure		Disease transmission by ungulates, wildlife attacks
(7) Biophysical external disturbances on physical public infrastructure		Colonization of roads by shrubs
*Socioeconomic external disturbances*		
(8) Socioeconomic external disturbances on resource		Pasture costs
(8) Socioeconomic external disturbances on external resource		Livestock feed costs
(8) Socioeconomic external disturbances on resource users		Illegal practice of the profession, subsidy supply shortage
(8) Socioeconomic external disturbances on private infrastructure*		Uncertain profitability of livestock products
*Social internal disturbances*		
(9) Internal disturbances on resource and resource users**		Labor shortage, weak generational renewal

Following the nomenclature proposed by Anderies et al. (2004), we used letters and numbers to name the components and links of the CIS framework. We used capital letters to name the system’s components (i.e., *A*: resource; *B*: resource users—hereinafter referred to as farmers; *C*: public infrastructure providers—hereinafter referred to as government; *D*: public infrastructure; *E*: external disturbances; *I*: internal disturbances). Additionally, we added the first three letters in lowercase to indicate the nature of the main components (*Aext*: External resource; *Bpri*: Private infrastructure; *Dsoc*: Social public infrastructure; *Dphy*: Physical public infrastructure; *Ebio*: Biophysical external disturbances; *Esoc*: Socioeconomic external disturbances; *Isoc*: Social internal disturbances) (Fig. 3; Table 1). The links were numbered from 1 to 9. We indicated the direction of the link by adding the letters of the components involved following the direction of the interaction to the number of the link (e.g., 1AB indicates an effect from resource to farmers, while 2CB indicates an effect from government to farmers) (Fig. 3; Tables 2 and 3).

#### Data coding

Each vulnerability was coded at two levels, from less detailed to more detailed: (1) component level and (2) nature of the vulnerability level. For instance, at the component level, high fuel and animal feed costs were both coded as a socioeconomic external disturbance harming the external resource (i.e., 8EsocAext). We then added additional information about the nature of the vulnerability (i.e., 8EsocAext-E and 8EsocAext-F, for fuel cost and animal feed cost, respectively) at a more detailed level to this code (see Table S2 for a complete list of vulnerabilities identified by farmers).

Three of the authors (ZMR, JASZ, IPI) were responsible for coding the farmers’ responses according to the following scheme: After an initial calibration, ZMR conducted a first round of coding of all the responses. The different types of coding were subsequently reviewed by IPI and, in case of disagreement, an alternative coding was annotated and discussed with the other authors (JASZ, ZMR) and reviewed by ZMR. This process was repeated until there were no more discrepancies. Once the coding of all responses was completed, the data were translated into English during the preparation of this manuscript for analysis and reporting.

Finally, we estimated the potential impact of each identified vulnerability on the maintenance of livestock farming systems by considering the percentage of farmers who mentioned it. For instance, if high feed costs (i.e., 8EsocAext-F) were mentioned by 100 farmers out of 255, the value assigned to this vulnerability is 39.2%.

### Social and ecological factors responsible for livestock farming systems vulnerabilities

To respond to objective 2, we carried out four different redundancy analyses (RDA) to summarize the interlinkages between the vulnerabilities perceived by farmers at the more detailed level of codification (i.e., at the nature level; Table S2), and a set of 23 explanatory variables describing the main components of the CIS framework (Table S3). The explanatory variables for each component are: Resource (A) availability as determined by the Normalized Difference Vegetation Index (*NDVI*) from 19 years of MODIS satellite imagery (2001 to 2019). This average NDVI was used as a proxy for the primary productivity and biomass of herbivore food resources at the location of each surveyed farming area. Farmers (B) were characterized using sociodemographic characteristics of the surveyed farmers (i.e., *Age*, experience as a farmer (*Exp*), gender (*Male, Female*), practice of transhumance or not (*Transh*)). In addition, the private infrastructure of farmers (Bpri) were characterized by the trend in the number of livestock heads (*HeadsTrend*) and farms (*FarmsTrend*) in the last decade at the municipality level; and by the farmer’s response to the number of animals owned and livestock management (i.e., *Sheep*, *Goats*, *Cattle*, selling other products beyond meat production or not (*Other_Prod*)). The location of the farm at the regional level (*CM, PY, CS, CA, NM, FU*; see Fig. 2 for the location of the study areas) was used to characterize the different governments (C). The human footprint (Venter et al. 2016), (i.e., an indicator of human population pressure, human land use, infrastructure, and human access (*Footprint*)), as well as the presence of protected areas in the farm surroundings (*PA*), was used as a proxy for the number and status of public infrastructure (D). Biophysical external disturbances (Ebio) were represented by the presence of large predators, (i.e., brown bear *Ursus arctos* and/or wolf *Canis lupus* (*Pred*)), vertebrate richness (*VerRich*), and the climatic conditions of the farmland surroundings (i.e., mean annual temperature (*Temp*), temperature seasonality (*Temp_sd*), mean annual precipitation (*Prec*), precipitation seasonality (*Prec_cv*)). Socioeconomic external disturbances (Esoc) were characterized by the importance of the European Union’s Common Agricultural Policy subsidy payments at each study site (*CAP, CAPpasture*). Table S3 shows the description and details of the calculations of each variable, and Tables S4 and S5 provide details (i.e., percentage, mean, standard error).

The first RDA included the variables as descriptors of the components of the system not composed by humans (resource and public infrastructure), i.e., (A) Resource and (D) Public infrastructure; the second RDA included the components of the system composed by humans (farmers and government), i.e., (B) Resource users and (C) Public infrastructure providers; the third RDA included the Private infrastructure of farmers (Bpri); and the fourth RDA included the external disturbances (E) to the systems, both of a biophysical and a socioeconomic nature. This organization of the RDA allowed us to compare the contribution of internal and external variables of the livestock farming systems to the vulnerabilities perceived by surveyed farmers. In the database each row represents a farmer (*n* = 255) and each column an explanatory variable describing the components of the CIS framework based on the response of the surveyed farmers (Table S4) and the location of the survey (Table S5). We used the vulnerabilities perceived by farmers at the nature level (Table S2) as a dichotomous response variable (i.e., 1 if the vulnerability was perceived by each farmer or 0 if it was not) and the variables explaining the nature of the vulnerabilities (Tables S3, S4, and S5) as explanatory variables. All continuous explanatory variables were log-transformed. We performed analyses using the *rda* function in the *vegan* package (Oksanen et al. 2019). The *rda* function computes a redundancy analysis of a matrix of response variables (24 vulnerabilities perceived by farmers; Table S2), constrained by a matrix of explanatory variables (23 variables; Tables S3, S4, and S5). The percentage of variance explained by each of the four groups of explanatory variables (one for each RDA) was used to identify the most important factors determining the association between perceived vulnerabilities and the variables. We used ANOVA with a Holm correction (999 permutations) to correct for multiple testing to determine whether the global model as well as each axis and explanatory variable were independently significant. All analyses were run in R (R Core Team 2020).

## Results

### Vulnerabilities

**Fig. 4:**
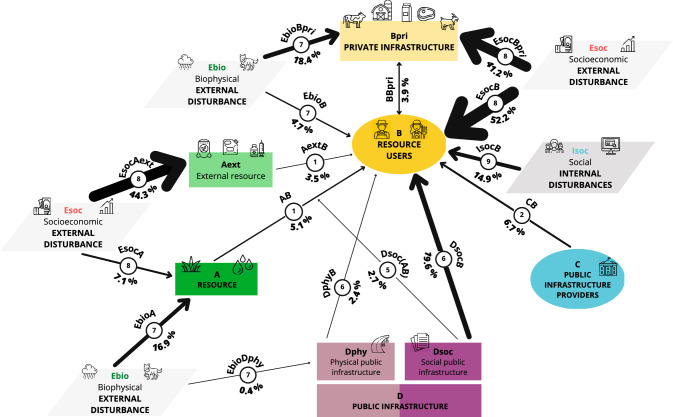
Representation of the vulnerabilities identified by the surveyed farmers in Spanish traditional livestock farming systems. The thickness of the arrows and the numbers next to the arrows indicate the degree to which each vulnerability mentioned by farmers is likely to affect the sustainability or maintenance of livestock farming systems, based on the percentage of farmers who mentioned each vulnerability. Coding examples of each vulnerability are listed in Table S2. See Figs. S2, S3 and S4 for individual representations of each study area

Figure 4 summarizes the vulnerabilities identified by the surveyed farmers represented in a CIS framework (see Table S2 for a complete list of vulnerabilities and coding examples). In total, farmers identified 24 vulnerabilities (i.e., 24 links at the nature level). Each farmer mentioned an average of 2.4 ± 1.2 (± standard deviation) vulnerabilities at the component level and 2.6 ± 1.3 (SD) vulnerabilities at the nature level, with a maximum of 7 vulnerabilities (one farmer), and six farmers did not mention any vulnerabilities (Table S6). See Appendix S3, Table S7 and Figures S2, S3 and S4 for a geographical analysis of the nature of the vulnerabilities in the six study areas.

Most farmers (78.8%, links 8EsocA, 8EsocAext, 8EsocB, 8EsocBpri) mentioned socioeconomic external disturbances as the main vulnerability for their livestock farming system, as they were directly mentioned to harm the farmers (link 8EsocB), the external resources (link 8EsocAext), the private infrastructure (link 8EsocBpri) and the resource (link 8EsocA). Socioeconomic external disturbances mainly harmed the farmers (52.2%, link 8EsocB) due to scarcity, uncertainty and/or inequality in the distribution of agricultural subsidies (31.8%, link 8EsocB-S), and the production and commercialization of livestock products (19.6%, link 8EsocB-M). Socioeconomic external disturbances also harmed the external resources (44.3%, link 8EsocAext) due to livestock feed costs (44.3%, link 8EsocAext-F) and the private infrastructure of the farmers (41.2%, link 8EsocBpri) mostly due to low profitability and decrease or uncertainty in profitability of livestock products (41.2%). Socioeconomic external disturbances also harmed the resource (7.1%, link 8EsocA) mainly due to high pasture costs (6.3%, link 8EsocA-P) (Fig. 4; Table S2).

Farmers mentioned biophysical external disturbances damaging the private infrastructure (link 7EbioBpri), the resource (link 7EbioA), the farmers (link 7EbioB), the physical public infrastructure (link 7EbioDphy) as the second main vulnerability to their farming systems (33.7%, links 7EbioA, 7EbioB, 7EbioBpri, 7EbioDphy). Biophysical external disturbances mainly harmed farmers’ private infrastructure (18.4%, link 7EbioBpri) and the resource (16.9%, link 7EbioA), principally due to wildlife attacks (18.0%) and rainfall shortages (6.7%), respectively. Also, biophysical external disturbances harmed the farmers (4.7%, link 7EbioB), mostly due to inclement weather experienced by farmers, and the physical public infrastructure (0.4%, link 7EbioDphy) through the colonization of roads by shrubs (Fig. 4; Table S2).

Other mentioned vulnerabilities were associated with the link between social public infrastructure and farmers (link 6DsocB) or the link between resources and farmers (link 5Dsoc(AB)) due to legal requirements that farmers must follow (22.0%, links 5Dsoc(AB), 6DsocB), such as bureaucratic problems (8.2%). Farmers also mentioned social internal disturbances harming the farmers (14.9%, link 9IsocB), such as working as a farmer is a demanding job (8.6%) or weak generational renewal (2.8%) (Fig. 4; Table S2).

The link between government and farmers (6.7%, link 2CB) indicates, among other things, the lack of government support for the pastoral activity (3.1%). The link between resources and farmers (5.1%, link 1AB) was mostly related to pasture shortages, whereas the link between external resources and farmers (3.5%, link 1AextB) was perceived as a vulnerability mainly because of livestock feed importation (2.7%). The link between farmers and their private infrastructure (3.9%, link BBpri) was mainly considered as a vulnerability due to the negative impact of livestock diseases (3.1%). The link between physical public infrastructure and farmers (2.4%, link 6DphyB) was indicated as a vulnerability due to the lack or poor condition of roads (Fig. 4; Table S2).

### Factors contributing to the vulnerabilities

**Fig. 5:**
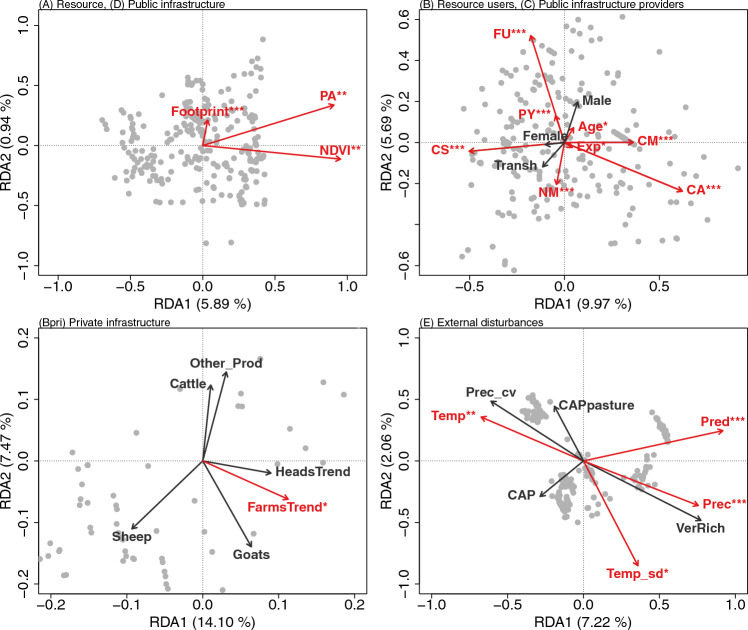
The ordination biplot of the four different redundancy analyses (RDA) conducted shows the relationship between the vulnerabilities identified by farmers (gray points; see Table S2 for complete list of vulnerabilities) and the explanatory variables (red and black arrows; see the Materials and Methods section and Table S3 for descriptions of the explanatory variables) related to the non-human components (top-left panel), the human components (top-right panel), the private infrastructure of the resource users (bottom-left panel), and the external disturbances (bottom-right panel). Red text and arrows show statistically significant relationships (significance codes: ***p ≤ 0.001; **p ≤ 0.01; *p ≤ 0.05). See the Materials and Methods section for details on statistical analysis and Table S8 for summary statistics and details of the results

The RDA revealed statistically significant associations between the farmers’ perceived vulnerabilities and variables that explain the nature of these vulnerabilities (Fig. 5; Table S8). The biplots of the RDA analysis (Fig. 5) illustrate these relationships across four key dimensions of the CIS framework: non-human components, human components, private infrastructure of resource users, and external disturbances.

Regarding the non-human components, which include resources and the public infrastructure (6.8% of the total variance; Fig. 5 top-left panel), the main vulnerabilities were significantly associated with primary productivity (*NDVI*), the presence of protected areas (*PA*) and the human footprint size (*Footprint*). *NDVI* and *PA* demonstrated stronger associations with perceived vulnerabilities (*p* ≤ 0.001), while *Footprint* indicated a weaker yet significant relationship (*p* ≤ 0.01). This suggests that landscape characteristics and conservation measures play a crucial role in shaping farmers' perceptions of vulnerability.

Concerning the human components, which encompass the farmers and the government (21.6% of variance; Fig. 5 top-right panel), vulnerabilities were mainly related to the location of the farm at the regional scale (*CM; PY; CS; CA; NM; FU; p* ≤ 0.001), but also to the age and experience level of the farmer (*Age; Exp; p* ≤ 0.05). Gender (*Female*; *Male*) and transhumance practices (*Transh*) did not show significant associations. These findings indicate that regional differences and certain farmer socio-demographics are key factors influencing vulnerability perceptions.

With regards to the private infrastructure of the farmers (15.7% of variance; Fig. 5 bottom-left panel), vulnerabilities were linked to the trend in the number of farms at the municipality level (*FarmsTrend*; *p* ≤ 0.05). Factors such as livestock numbers (*Sheep*, *Goats*, *Cattle*) and product diversification (*Other_Prod*) did not show significant associations. This suggests that broader local farm trends may be more influential in shaping vulnerability perceptions than individual farm characteristics.

In terms of external disturbances (9.3% of variance; Fig. 5 bottom-right panel), vulnerabilities were associated with both climatic factors, including mean temperature (*Temp*; *p* ≤ 0.01), temperature seasonality (*Temp_sd*; *p* ≤ 0.05), and annual precipitation (*Prec*; *p* ≤ 0.001), as well as the presence of large predators (*Pred*; *p* ≤ 0.001). Socioeconomic external disturbances such as CAP subsidies (*CAP*; *CAPpasture*) did not show significant associations. These results highlight the importance of environmental factors in farmers' risk assessments, suggesting that climate variability and conflicts with wildlife may be perceived as more immediate challenges compared to the support provided by economic policy instruments like the CAP.

## Discussion

In this study, we disentangled the major vulnerabilities identified by Spanish farmers across different geographical areas, which were primarily caused by external socioeconomic and biophysical disturbances, such as resource costs, low profitability of livestock products, climate variability, and conflicts with wildlife. We then highlighted the key factors that contribute to these vulnerabilities, noting that their variability largely depended on the overall primary productivity of the farmer´s region, the location of the farm, the presence of large predators and climatic conditions (mostly rainfall).

While our study primarily focused on the vulnerabilities of extensive livestock farming systems in Spain, our findings have important implications for broader concepts of food security and ecosystem services. The socioeconomic vulnerabilities identified by farmers, such as uncertainties in subsidies and market challenges, directly impact local and regional food security by affecting the stability and accessibility of livestock products (Michel-Villarreal et al. 2019). Moreover, extensive livestock systems contribute to the provision of ecosystem services such as maintaining biodiversity, preventing wildfires through grazing, and preserving cultural heritage (Ronchi and Ramanzin 2024). The vulnerabilities we identified, especially those related to biophysical external disturbances such as climate change and conflicts with wildlife, pose significant threats to the continued provision of these services. Our findings thus highlight the complex interplay between extensive livestock farming, food security, and ecosystem services, underscoring the need for integrated approaches to support these traditional systems while ensuring their multifunctional benefits to society and the environment.

### Vulnerabilities associated with socioeconomic conditions

Our results show that socioeconomic external disturbances associated with farming intensification processes are the main vulnerabilities perceived by farmers. Key issues included uncertainty in agricultural subsidies, challenges in production and commercialization of livestock products, and rising livestock feed costs. These findings reflect the broader transition in Spain from a family subsistence economy to a market economy, with the loss of economic profitability being considered one of the main reasons for the decline of traditional livestock practices such as transhumance (Oteros-Rozas et al. 2013; Fernández-Giménez and Ritten 2020).

Our study's emphasis on socioeconomic vulnerabilities aligns with global trends in the intensification of livestock production systems (Cortner et al. 2019). For instance, in Mongolia, semi-nomadic pastoralism was replaced by sedentary pastoralism in the 1960s, which is currently being intensified (Briske et al. 2015). Similarly, in East Africa, mixed crop–livestock farms are undergoing a process of intensification (Kindu et al. 2014). In the European Mediterranean basin, intensification and extensification processes have also affected livestock production in southern countries over the last decades, particularly in light of the marginalization of mountain areas and the resulting socioeconomic and environmental changes (Jiménez-Olivencia et al. 2021). While these global trends provide context, our findings offer a more nuanced understanding of the Spanish context. Specifically, our results highlight the specific economic pressures faced by farmers, from subsidy distribution to feed costs, offering a comprehensive view of the challenges in the Spanish livestock sector.

Our study highlights the high costs of external sources of livestock feed as an important vulnerability, especially in the more arid regions where pasture is scarce. This finding is crucial as it reflects a broader trend in drylands, where supplementary feed has become a widely used strategy to combat harsh climatic conditions (Rjili et al. 2023). However, this method may be environmentally and socio-economically problematic. We found that farmers' concerns about feed costs may be closely linked to issues of land degradation and economic sustainability. This aligns with previous research indicating that supplementing livestock with external feed resources may result in land degradation due to factors such as maintaining large livestock numbers, which reduces the carrying capacity of pastures (Ibañez et al. 2014; Rjili et al. 2023). Moreover, our findings on the economic pressures faced by farmers are consistent with studies showing that dependence on supplementation clearly increases costs for livestock producers (Schulze et al. 2016). Our results are particularly relevant when considering specific regional contexts. For example, on the island of Fuerteventura, the pasture shortage associated with aridity was compounded by a high dependence on fodder importation from mainland Spain (Banos-González et al. 2016). The vulnerabilities identified in our study extend beyond local contexts, as evidenced by the recent global impact of the war in Ukraine on cereal availability (Gross 2022). Based on our findings and the existing literature, we suggest that increasing local self-sufficiency (i.e., locally produced feed) in animal feed and reducing dependence on external inputs could together decrease the vulnerability of these farming systems while improving their sustainability (Catarino et al. 2021). However, achieving this would require overcoming barriers such as environmental constraints on feed production and the need to balance herd size with profitability.

Our results indicate the low profitability of livestock products and problems associated with the production and commercialization of livestock products also arose as one of the main concerns of farmers. This aligns with the broader trend of increasing economic pressures on traditional farming systems, where market dynamics often favor more intensive production models. Although global demand for livestock products has risen (Fukase and Martin 2020), the opportunity for economic development in extensive farming systems is limited by the dominance of intensive livestock farming systems, which has been growing steadily over the last decades (Clay et al. 2020). This shift toward intensification has had numerous negative impacts on the environment, human health, safety and animal welfare (Goldberg 2016; Smit and Heederik 2017). Our findings highlight farmers’ frustrations with these pressures, which echo concerns about the need for more sustainable production systems. In response to these issues, some researchers advocate for a transition toward plant-forward diets to reduce environmental impacts (Kim et al. 2020; Pieper et al. 2020), while others suggest that public attitudes toward animal welfare and sustainability could be important motivators for reducing meat consumption and promoting human health (Clark et al. 2016). Nevertheless, other studies argue that healthy, sustainable and ethical diets are possible under several livestock production methods and cultural contexts and, thus, restricting animal-source foods may increase food system vulnerabilities (Leroy et al. 2022). Measures such as marketing high value-added products (Martínez et al. 2023) and promoting short-distance transport of products, such as local consumption and short marketing channels (Pedersen et al. 2018), could help boost profitability while reducing the environmental impacts of livestock farming (Briske et al. 2015). For example, in Fuerteventura, the region where farmers reported the fewest problems in production and commercialization, more than 90% have diversified their farm products, offering items like milk and cheese alongside meat.

Our results reveal that farmers identified the scarcity, uncertainty, dependency, and inequitable distribution of government subsidies as key vulnerabilities affecting their livestock farming systems. This widespread concern highlights a clear dependency on external subsidies, which, according to the farmers, are not distributed equitably or efficiently. These findings support recent calls to transform the European Union’s CAP to better address sustainability challenges (Pe’er et al. 2020). In particular, our results align with the growing demand for urgent CAP legislation reform aimed at guaranteeing access to sufficient, safe, sustainable and nutritious food for Europe (Recanati et al. 2019). Nevertheless, our research also highlights how the substantial financial resources of the CAP may have been misused, failing to adequately support the achievement of the Sustainable Development Goals (Scown et al. 2020). To address these shortcomings, our findings reinforce the need for agricultural subsidy reform, focusing on healthier and more sustainable food systems (Springmann and Freund 2022). This could involve promoting a shift toward organic practices or maintaining traditional self-sufficient farming systems based on the sustainable use of local resources, such as extensive livestock systems, encouraging the coexistence of traditional practices with biodiversity conservation (Crespin and Simonetti 2019; Aguilera-Alcalá et al. 2022). In summary, our results emphasize the urgency of rethinking the distribution of agricultural subsidies to more effectively support the economic and environmental sustainability of extensive livestock farming systems that heavily rely on these aids.

Our findings reveal that farmers identified various social vulnerabilities, such as the bureaucracy associated with institutions and social internal vulnerabilities like hard working conditions and labor shortages, as significant challenges. However, contrary to previous studies that emphasize the importance of generational renewal and the low social recognition of the profession as major sustainability concerns in extensive livestock systems (Bernués et al. 2011; Oteros-Rozas et al. 2013; Wolff 2024), generational renewal was surprisingly not highlighted as a primary vulnerability in our study areas, particularly in the Pyrenees and Murcia. Although farmers were encouraged to discuss general problems and problems for the future, they may have focused on talking about the current situation of their farms and not on thinking about the future. Understanding the factors that contribute to generational renewal is essential for the long-term viability of these farming systems. Recent studies have identified personality traits, early involvement in farming, career path and individual perceptions of farming as key elements influencing generational renewal in Europe (Coopmans et al. 2021). This aligns with our observation that farmers may focus more on current challenges, highlighting the need for awareness and discussion around the future of their farms. In addition, the bureaucratic obstacles faced by farmers in our study resonate with findings from other EU countries (Whitton and Carmichael 2024). To mitigate these bureaucratic burdens, policies should aim to improve coordination among institutions and streamline funding processes, enabling farmers to focus on sustainable practices. Additionally, programs like farmer schools and formal education courses in product marketing could play a crucial role in fostering generational renewal and supporting traditional livestock practices (Oteros-Rozas et al. 2013; Góngora et al. 2019). For example, in Cazorla, a combination of economic incentives supported by European and regional governments and the establishment of a school for young farmers has successfully increased generational renewal, making this area home to the largest population of transhumant sheep in Western Europe (Aguilera-Alcalá et al. 2022; Velamazán et al. 2024).

### Vulnerabilities associated with environmental conditions

Our study reveals that farmers were concerned about vulnerabilities associated with the harm of biophysical external disturbances to resources and, to a lesser extent, the farmers, due to extreme and harsh weather conditions. This aligns with scientific projections indicating an increase in extreme weather events (Fischer et al. 2021). Farmers in the most arid study areas (Cazorla, Murcia, Fuerteventura) considered climatic conditions (mainly lack of rainfall and drought) as critical factors impacting the sustainability of their livestock farming systems. These perceptions corroborate the literature, which suggests that climate anomalies are expected to have a negative impact on the sustainability of the livestock farming systems, especially in the Mediterranean basin (Rojas-Downing et al. 2017). The projected climatic conditions (e.g., less frequent rainfall) could lead to a greater dependence on external food inputs (i.e., supplementary feed). This dependence poses important socioeconomic challenges, as highlighted in previous research (Thomasz et al. 2020). Interestingly, while the potential impacts of climate change on livestock health have been documented (Godde et al. 2021), this vulnerability did not emerge as a concern among farmers in any of the study areas. However, our study revealed that farmers are increasingly concerned about climatic disturbances and their impact on traditional practices. While traditional practices like transhumance are generally well adapted to recurrent climate oscillations, our results indicate that these practices are being challenged by climatic anomalies associated with climate change. This discrepancy suggests a need for increased awareness and dialogue around the broader implications of climate change on livestock health within these communities. These findings align with other regions, such as the Himalayas, where similar challenges have been observed (Aryal et al. 2014).

Our study also underscores the importance of local livestock breeds in climate adaptation strategies. This finding is consistent with broader recommendations for including livestock breed conservation in climate adaptation plans (Sejian et al. 2015). Moreover, our results suggest that such adaptation plans could potentially increase livestock productivity and, consequently, pastoralists’ profits, as observed in other contexts (Lamy et al. 2012). The farmers in our study particularly highlighted the Segureña sheep bred in Cazorla as an example of a native breed well adapted to the local territory and capable of optimizing production in difficult environments (Velamazán et al. 2024). This aligns with previous research on the breed's adaptability and productivity (Rubio and Roig 2017).

Furthermore, our findings indicate that certain livestock husbandry practices could lead to increased carbon sequestration and contribute to the fight against climate change by reducing greenhouse gas emissions, such as practices related to grazing management or practices that enhance forage production and avoid emissions associated with feed production (Herrero et al. 2016). Furthermore, our study reinforces the ecological importance of traditional practices like transhumance. Farmers noted the role of transhumant livestock in facilitating long-distance seed dispersal along drove roads, a finding that aligns with recent ecological research (García-Fernández et al. 2019).

Our findings indicate that farmers are increasingly concerned about the emerging conflict between wildlife and humans, mainly in relation to wildlife attacks on livestock, and to a lesser extent, wild boar rooting or grazing competition with wild ungulates. Although these vulnerabilities were less frequently highlighted compared to socioeconomic external disturbances, they still pose significant challenges in certain regions. For instance, in the Cantabrian Mountains and the Central System, conflicts with large predators like wolves and bears were prominent, while in the Pyrenees, farmers cited issues with vultures. Also, in the Cantabrian Mountains, farmers expressed concerns over resource-related wildlife impacts, especially due to pastures being affected by wild boar rooting and due to grazing competition with wild ungulates. These findings complement broader trends in Europe, where both carnivore and wild ungulate populations are increasing through passive rewilding processes (Chapron et al. 2014; Valente et al. 2020). As such, farmer-wildlife interactions are expected to increase, particularly in human-dominated landscapes (e.g., Pascual-Rico et al. 2020). Despite this, the long-term sustainability of livestock farming systems seems to be more threatened by socioeconomic external disturbances, which were the primary concern for farmers across most study areas. Only in the Cantabrian Mountains did biophysical external disturbances, such as wildlife conflicts, surpass socioeconomic concerns.

In general, the perception of Spanish society, and farmer perceptions in particular, about the ecosystem services provided by scavengers is positive (Morales-Reyes et al. 2018; Aguilera-Alcalá et al. 2020). However, there is a possibility of generating social alarm about attacks on livestock attributed to carnivores due to media coverage or from viral dissemination of partial and biased information through social media (Delibes-Mateos 2020). Interestingly, some farmers reported incidents involving vultures, which may lead to misunderstandings and promote or reinforce negative perceptions of wildlife (Lambertucci et al. 2021). Consequently, such negative perceptions could lead to harmful actions that seriously compromise biodiversity conservation, for example, through illegal actions such as the use of poison (Mateo-Tomás et al. 2020). Our results underscore the need for better public education and clearer communication to prevent the spread of misinformation and mitigate the risk of negative actions against wildlife. Additionally, policies that promote coexistence between livestock and wildlife are crucial. Addressing these concerns is essential for both the sustainability of livestock farming and wildlife conservation. Based on our results, we argue that it is important to communicate to the public the ecosystem services that both mammalian carnivores and vultures provide to society, as suggested by recent studies (Lozano et al. 2019; García-Jiménez et al. 2022). This approach could help counteract the negative views expressed by farmers in our study. Moreover, farmers’ perceptions of vulnerabilities may be shaped by the values they attach to natural assets. Recent research (Klebl et al. 2024) suggests that farmers who prioritize the instrumental benefits of biodiversity tend to focus more on socioeconomic challenges, while those who recognize biodiversity's intrinsic value may lean toward more holistic management approaches. Understanding these value-based perspectives can offer deeper insights into how vulnerabilities are perceived and addressed, highlighting the complexity of the human-wildlife relationship.

Our research highlights the need for innovative initiatives to address these conflicts. For instance, the Foundation for the Conservation of the Bearded Vultures (FCQ) created a special certification (“Pro-Biodiversidad”) to support the extensive livestock sector, while improving the conservation of scavengers in the Picos de Europa National Park. This aligns with the needs expressed in our study for support in coexisting with wildlife. Similarly, the “Grazing with Wolves” initiative^1^ promotes the coexistence between sheep farming and wolves, directly addressing the concerns raised by our participants regarding predator conflicts. In addition, the effectiveness of livestock husbandry systems (e.g. attentive herding during the day, livestock guardian dogs, fencing at night) has been shown to mitigate predation on livestock (Pimenta et al. 2017; Durá-Alemañ et al. 2024). However, our findings also suggest that current monetary compensation programs in Spain may not be sufficiently addressing farmers' concerns, echoing studies from other Mediterranean countries that question the effectiveness of such programs. In contrast, other common tools promoted in Spain to mitigate conflicts such as monetary compensation programs, do not seem to be an effective conservation tool in other Mediterranean countries (Bautista et al. 2019). Importantly, our research underscores the interconnectedness of traditional livestock farming and biodiversity conservation. The farmers we interviewed expressed concern about the potential ecological impacts of abandoning traditional practices. This aligns with studies showing that the disappearance of traditional livestock farming systems could also lead to associated impacts on the conservation of animal and plant biodiversity (Carmona et al. 2013; Aguilera-Alcalá et al. 2022).

## Conclusion

Traditional extensive livestock farming systems in the European Mediterranean region are rapidly declining, despite institutional efforts, which threaten essential ecosystem services. This study provides the first large-scale examination of livestock farmers' perceptions across extensive farming systems in Spain regarding the primary sources of vulnerability affecting these systems. Our findings underscore the usefulness of combining the study of CIS framework with farmers’ social perceptions to better understand the major vulnerabilities faced by traditional extensive livestock farming systems. Key vulnerabilities perceived by farmers primarily include socioeconomic and biophysical external disturbances affecting farmers, external resources, private and public infrastructures, and the resource. Factors such as primary productivity, farm location, the presence of large predators and climatic conditions are viewed as the main drivers of these vulnerabilities. While our study relies on perceived rather than objective vulnerabilities, these insights can guide policy development aimed at improving farmers' well-being and enhancing social-ecological resilience.

Future policy recommendations should focus on increasing social recognition of the sector, supporting farmers through public administration initiatives, improving the quality and promoting the differentiation of livestock products, and fostering coexistence between farmers and wildlife. We propose allocating Common Agricultural Policy (CAP) funds not only to support pastoral practices conducive to farmers-wildlife coexistence but also to enhance farm profitability through market differentiation and improving access to essential resources. Supporting rural infrastructure, strengthening farmers' resilience to climate variability, and fostering adaptive management plans will be crucial to addressing both internal and external vulnerabilities. These measures are critical for addressing the major internal and external vulnerabilities that livestock farmers face, particularly in the context of global challenges such as farming intensification, climate change, and rewilding.

While our study provides valuable insights into the vulnerabilities of extensive livestock farming systems in Spain, we acknowledge its limitations. Since the data used reflect a specific period, concerns about its current relevance may arise given the dynamic nature of agricultural systems and global challenges. However, the alignment of our findings with more recent assessments and ongoing research supports the continued applicability of our conclusions. Future studies should aim to capture more recent shifts and emerging challenges to ensure that policy recommendations remain timely and effective. Looking forward, further research should explore the intricate relationships between these vulnerabilities and the ongoing decline of extensive farming systems, as well as how these vulnerabilities interact with global challenges to formulate effective policies that ensure long-term social, economic, and environmental sustainability.

## Supplementary Information

Below is the link to the electronic supplementary material.

Supplementary file1 (PDF 1382 KB)

## Data Availability

To safeguard participants’ privacy and comply with the General Data Protection Regulation of the European Union (Regulation EU 2016/679) and Spanish law (Organic Law 3/2018, of December 5, on the Protection of Personal Data and the Guarantee of Digital Rights), we are unable to make the raw data used in the study publicly available. However, relevant coding examples from the interviews are available in the Supplementary Material.

## Footnotes

1 http://www.pastandoconlobos.com
