# Supplementary material for: Farmer perceptions of the vulnerabilities of traditional livestock farming systems under global change
Source: Ambio. 2025 Feb 26;54(8):1353–71. doi: 10.1007/s13280-025-02150-8 (PMC12214157; doi:10.1007/s13280-025-02150-8)
Supplement: Supplementary file 1 — Supplementary file1 (PDF 1382 KB) [file 13280_2025_2150_MOESM1_ESM.pdf]

Ambio

Supplementary Information

This supplementary information has not been peer reviewed.

Title: **Farmer perceptions of the vulnerabilities of traditional livestock farming systems under global change**

Authors: Zebensui Morales-Reyes, Jomar M. Barbosa, José A. Sánchez-Zapata, Irene Pérez-Ibarra

This material includes:

Appendix S1. Calculation of representative sample sizes.

Appendix S2. Details on data collection.

Appendix S3. Geographical analysis of the nature of the vulnerabilities.

Table S1. Population size, sample size, and margin of error in each study area.

Table S2. Complete list of vulnerabilities identified by the surveyed farmers as coded by links between the main components of the Coupled Infrastructure System.

Table S3. Overview of the response and explanatory variables used in the redundancy analyses (RDA).

Table S4. Total number of surveys conducted and summary of explanatory variables obtained from surveys of the farmers for the set of study areas and in each study area.

Table S5. Summary of explanatory variables collected from the geographic information system for all study areas and by each study area.

Table S6. Number of vulnerabilities identified by surveyed farmers by each study area and for all study areas at the component and the nature of the vulnerability levels.

Table S7. Complete list of vulnerabilities identified by the surveyed farmers by each study area.

Table S8. Summary statistics and results of the four different redundancy analyses conducted showing the relationship between the vulnerabilities identified by farmers and the explanatory variables describing the components of the Coupled Infrastructure System.

Fig. S1. Evolution of the number of livestock (sheep, goats, cattle, and pigs) in the last three decades (1994 - 2020) in Spain.

Fig. S2. Representation of the vulnerabilities identified by the surveyed farmers in the Cantabrian Mountains and the Pyrenees.

Fig. S3. Representation of the vulnerabilities identified by the surveyed farmers in the Central System and Sierras de Cazorla, Segura y Las Villas Natural Park.

Fig. S4. Representation of the vulnerabilities identified by the surveyed farmers in the northwest region of Murcia and Fuerteventura.

Appendix S1. Calculation of representative sample sizes.

We used Cochran's equation (1977) (Eq. 1), adjusted to finite populations (Bartlett *et al.* 2001) (Eq. 2), to calculate the size of representative samples in each of the study areas (see Table S1):

$$n_0 = \frac{Z^2pq}{e^2} \qquad \text{(Eq. 1)}$$

where n_0 is the sample size, Z^2 is the abscissa for the normal curve that cuts off an area α at the tails (1 – α equals the desired confidence level, e.g., for a confidence level of 95%, α is 0.05 and the critical value is Z^2= 1.96), *e* is the desired margin of error, *p* is the estimated proportion of an attribute that is present in the population, and *q* is 1-*p.* Since the *p* value in our population was unknown we used *p*=0.5, which is conservative and gives the largest sample size.

For finite populations, the sample size (n_0) was adjusted using the following equation:

$$n = \frac{n_0}{1+\frac{(n_0-1)}{N}} \qquad \text{(Eq. 2)}$$

where *n* is the sample size and *N* is the population size.

Cochran's equation (1977) is widely used in socio-ecological studies to calculate survey sample size (e.g., Heitz et al. 2009; Perez et al. 2012; La Rosa & Privitera 2013; Mekasha et al. 2014; Morales-Reyes et al. 2018). To calculate the sample size, we used 95% confidence level, margin of error between 10 and 15%, and *p* value of 0.5. The final margin of error was calculated using the following equation (Eq. 3):

$$e = \sqrt{\frac{N-n}{N-1}} * Z^2\sqrt{\frac{pq}{n}} \qquad \text{(Eq. 3)}$$

where *e* is the margin of error, *N* is the population size, *n* is the sample size, Z^2 is the abscissa for the normal curve that cuts off an area α at the tails, *p* is the estimated proportion of an attribute that is present in the population, and *q* is 1-*p*. $\sqrt{\frac{N-n}{N-1}}$ is the correction factor for finite populations.

Table S1 shows the total farmer population (i.e., population size; *N*), total number of surveys (i.e., sample size; *n*) and final margin of error of the survey sample in each study area.

Appendix S2. Details on data collection.

We designed a sampling strategy that consisted of three main stages: (i) for each study area, an initial set of extensively managed farms was randomly selected from the Spanish General Register of Livestock Farms; (ii) contact details of farmers in each study area with extensive livestock farms were obtained from the local sanitary authorities to ensure we could reach all selected participants; (iii) we met farmers on or near their farms to conduct the survey, aiming to capture their direct experiences and perceptions. Occasionally, in cases where access to some farmers was limited, we identified additional farmers by using the snowball sampling technique (i.e., we asked farmers to name other farmers in the area), a technique commonly used when conducting social research in biodiversity conservation and the study of social-ecological systems (e.g., Anadón et al. 2009; Newing 2010; Martín-López et al. 2011; Morales-Reyes et al. 2018).

The questionnaire was pre-tested on a small sample of farmers in the northwest region of Murcia to improve its readability and clarity. For each study area, we estimated a representative sample size of farmers at a 95% confidence level, with a sampling error ranging 10.6-15.1% depending on the study area (see Appendix S1 for additional details and Table S1 for the total farmer population and sample size in each study area). In all cases, we followed ethical standards of social surveys by informing respondents that their participation was voluntary and that we would ensure their anonymity.

Appendix S3. Geographic analysis of the nature of the vulnerabilities.

Figures S2, S3 and S4 shows the vulnerabilities perceived by farmers from the six studied areas (see Table S7 for a complete list of vulnerabilities and coding examples by each study area and Fig. 2 for the location of the study areas). The number of components in which farmers mentioned vulnerabilities and the nature of those vulnerabilities varied among the different study areas (Kruskal-Wallis test; component level: $\chi^2 = 15.5$, df = 5, P = 0.008; nature level: $\chi^2 = 14.5$, df = 5, P = 0.013). Farmers from the Central System mentioned the fewest vulnerabilities (i.e., 11 links at the nature level), whereas farmers from the Cantabrian Mountains mentioned the highest number of vulnerabilities (i.e., 21 links at the nature level). However, farmers from the Pyrenees mentioned the highest number of vulnerabilities on average (mean ± Standard Deviation = 3.2 ± 1.3 vulnerabilities at the nature level) (Table S6).

Socioeconomic external disturbances were considered by farmers to be the main concern in all study areas (>75%) except in the Cantabrian Mountains (52.5%), where biophysical external disturbances were the most frequently mentioned (72.5%). Socioeconomic external disturbances harming the resource users (link 8EsocB) and the private infrastructure of the resource users (link 8EsocBpri) were commonly mentioned by farmers as the main vulnerability for their farming system (25% - 72.7% depending on the study area). Socioeconomic external disturbances harming the external resources (link 8EsocAext) were more often mentioned in Fuerteventura (78.0%), Murcia (55.2%) and the Pyrenees (50.0%), whereas socioeconomic external disturbances harming the resources (link 8EsocA) were mainly mentioned in Cazorla (21.2%) and Murcia (13.8%) (Figs. S2, S3 and S4; Table S7).

Biophysical external disturbances that harmed farmers' private infrastructure (mostly wildlife attacks; link 7EbioBpri) were mainly mentioned by farmers from the Cantabrian Mountains (70.0%), the Central System (36.4%) and the Pyrenees (15.6%) as an important vulnerability for their farming system. In addition, biophysical external disturbances harming the resources (mostly extreme weather events but also wildlife-related disturbances; link 7EbioA) were particularly relevant in the Cantabrian Mountains (25.0%), mainly due to pastures being affected by wild boar rooting (22.5%). In Fuerteventura (25.4%), Murcia (17.2%) and Cazorla (15.2%), biophysical external disturbances harmed the resource (link 7EbioA), mainly due to drought and rainfall shortage (Figs. S2, S3 and S4; Table S7).

Vulnerabilities related to social public infrastructure (links 5Dsoc(AB) and 6DsocB) were largely mentioned in the northern areas (i.e., the Pyrenees (46.9%) and the Cantabrian Mountains (40.0%)). Vulnerabilities associated with social internal disturbances (link 9IsocB) were mainly mentioned by farmers from the Pyrenees (37.5%), Murcia (25.9%), and Cazorla (18.2%) (Figs. S2, S3 and S4; Table S7).

The link between public infrastructure providers and resource users (link 2CB) were mentioned as a vulnerability more often in the Pyrenees (15.6%). The link between external resources and resource users (link 1AextB) was only mentioned as a vulnerability by farmers from Fuerteventura

(15.3%), whereas the link between resources and resource users due to pasture shortages (link 1AB) was mainly mentioned as a vulnerability in the Cantabrian Mountains (10.0%), Cazorla (9.1%) and Murcia (6.9%). The link between resource users and their private infrastructure (mainly livestock diseases; link BBpri) was mainly mentioned as a vulnerability in the Pyrenees (9.4%) and Murcia (6.9%). The lack of roads or poor conditions of roads (link 6DphyB) was only mentioned as a vulnerability by farmers from the Cantabrian Mountains (10.0%) and Cazorla (6.1%), whereas the colonization of roads by shrubs (link 7EbioDphy) was only mentioned as a vulnerability in the Cantabrian Mountains (2.5%) (Figs. S2, S3 and S4; Table S7).

Table S1. Population size (*N*), sample size (*n*), and margin of error (in %) in each study area. Population size refers to farms with >25 head of sheep or goats, and >10 head of cattle or other livestock.

**Study area**	***N***	***n***	**Margin of error**
Cantabrian Mountains	246	40	14.2
Pyrenees	86	32	13.8
Central System	148	33	15.1
Sierras de Cazorla, Segura y Las Villas Natural Park	122	33	14.6
Northwest region of Murcia	176	58	10.6
Fuerteventura	287	59	11.4
**Total**	**1,065**	**255**	**5.4**

Table S2. Complete list of vulnerabilities identified by the surveyed farmers as coded by links between the main components of the Coupled Infrastructure System (see Fig. 3 and methods for details on data coding). Numbers in parenthesis indicate the number of surveyed farmers and percentage relative to the total of farmers (n=255) who identified each vulnerability. Vulnerabilities whose values were 5 - 15% are in italic and >15% in bold. See Table S7 for details at the nature of problem levels by each study area.

**Component level**		**Nature level**		
**Name**	**Description**	**Name**	**Description**	**Coding examples**
1AB (n=13; 5.10%)	Resource-Resource users	*1AB (n=13; 5.10%)*	Pastures-Farmers	Excess livestock (n=2; 0.78%), *pasture shortage (n=13; 5.10%)*
1AextB (n=9; 3.53%)	External resource-Resource users	1AextB (n=9; 3.53%)	Food input-Farmers	Cereal shortage (n=1; 0.39%), difficulties in growing pastures (n=1; 0.39%), food importation (n=7; 2.75%), high costs (n=2; 0.78%), low quality of animal food (n=2; 0.78%)
BBpri (n=10; 3.92%)	Resource users - Private infrastructure	BBpri (n=10; 3.92%)	Farmers-Farms	High investment (n=1; 0.39%), livestock diseases (n=8; 3.14%), livestock dung accumulation (n=1; 0.39%), livestock losses (n=1; 0.39%)
2CB (n=17; 6.67%)	Public infrastructure providers-Resource users	*2CB (n=17; 6.67%)*	Government-Farmers	Cheats and lack of trust in wildlife attacks (n=1; 0.39%), corruption and lack of information (n=1; 0.39%), ecologists (n=1; 0.39%), government´s lack of support to the sector (n=7; 2.75%), government´s lack of support to young farmers (n=1; 0.39%), low cooperation among farmers (n=1; 0.39%)
5Dsoc(AB) (n=7; 2.75%)	Social public infrastructure-(Resource-Resource users)	5Dsoc(AB) (n=7; 2.75%)	Institutions-(Farmers-Pasture management)	Economic resources shortage (n=1; 0.39%), limits and management (n=1; 0.39%), mountain and agricultural land management (n=1; 0.39%), not allowed to manage shrub colonization (n=2; 0.78%), pasture shortage (n=3; 1.18%)
6DphyB (n=6; 2.35%)	Physical public infrastructure-Resource users	6DphyB (n=6; 2.35%)	Roads-Farmers	Lack of roads (n=2; 0.78%), not allowed to create roads (n=2; 0.78%), poor conditions of roads (n=3; 1.18%)
**6DsocB (n=50; 19.61%)**	Social public infrastructure-Resource users	**6DsocB (n=50; 19.61%)**	Institutions-Farmers	*Bureaucracy (n=21; 8.24%),* cheats in number of livestock (n=2; 0.78%), lack of electric supply (n=1; 0.39%), lack of regulations (n=1; 0.39%), legal requirements for carcass disposal (n=2; 0.78%), legal requirements for livestock numbers (n=1; 0.39%), legal requirements for livestock sanitation (n=1; 0.39%), livestock sanitation poorly done (n=3; 1.18%), low monetary compensations (n=1; 0.39%), mandatory carcass removal insurance (n=7; 2.75%), many inspections (n=1; 0.39%), many legal requirements (n=3; 1.18%),

				more carcass removal options needed (n=1; 0.39%), restrictions to animal movements (n=1; 0.39%), restrictions to number of animals (n=1; 0.39%), sanitation (n=5; 1.96%), sanitation costs (n=3; 1.18%), uncertain compensations (n=1; 0.39%), very rigorous livestock sanitation (n=2; 0.78%)
**7EbioA (n=43; 16.86%)**	Biophysical external disturbances-Resource	*7EbioA-C (n=31; 12.16%)*	Climate-Pasture	Cereal shortage (n=1; 0.39%), difficulties in growing pastures (n=1; 0.39%), drought (n=11; 4.31%), inclement weather (i.e. long winter) (n=1; 0.39%), pasture shortage (n=5; 1.96%), *rainfall shortage (n=17; 6.67%),* supplementary feeding needed (n=1; 0.39%), water shortage (n=2; 0.78%)
		7EbioA-W (n=12; 4.71%)	Wildlife-Pasture	Grazing competence with ungulates (n=3; 1.18%), wild boar crop damage (n=1; 0.39%), wild boar rooting (n=11; 4.31%)
7EbioB (n=12; 4.71%)	Biophysical external disturbances-Resource users	7EbioB (n=12; 4.71%)	Climate-Farmers	Inclement weather (i.e. cold weather, excess rainfall, snowy weather, high temperature variation, hard winter, highland climate) (n=12; 4.71%), isolated people (n=1; 0.39%)
**7EbioBpri (n=47; 18.43%)**	Biophysical external disturbances-Private infrastructure	**7EbioBpri (n=47; 18.43%)**	Wildlife-Livestock	Disease transmission by ungulates (n=3; 1.18%), increase of wolves (n=1; 0.39%), **wildlife attacks (n=46; 18.04%)**
7EbioDphy (n=1; 0.39%)	Biophysical external disturbances-Physical public infrastructure	7EbioDphy (n=1; 0.39%)	Natural vegetation-Roads	Colonization of roads by shrubs (n=1; 0.39%)
8EsocA (n=18; 7.06%)	Socioeconomic external disturbances-Resource	*8EsocA-P (n=16; 6.27%)*	Markets-Pastures	Cannot improve facilities (n=1; 0.39%), *high costs (n=16; 6.27%)*
		8EsocA-W (n=2; 0.78%)	Markets-Water	Difficulties in growing pastures (n=2; 0.78%), high costs (n=2; 0.78%)
**8EsocAext (n=113; 44.31%)**	Socioeconomic external disturbances-External resource	8EsocAext-E (n=9; 3.53%)	Markets-External input	Cost increase (n=2; 0.78%), high costs (n=7; 2.75%)
		**8EsocAext-F (n=113; 44.31%)**	Markets-Food input	Cost increase (n=7; 2.75%), **high costs (n=106; 41.57%)**, prices uncertainty (n=1; 0.39%)
**8EsocB (n=133; 52.16%)**	Socioeconomic external disturbances-Resource users	8EsocB-C&T (n=5; 1.96%)	Credits & Taxes-Farmers	Cost increase (n=1; 0.39%), credit shortage (n=1; 0.39%), high costs (n=2; 0.78%), high interests (n=1; 0.39%), many taxes (n=1; 0.39%)
		8EsocB-E (n=4; 1.57%)	Economic crisis-Farmers	Economic crisis (n=4; 1.57%)
		8EsocB-F (n=4; 1.57%)	Free riding-Farmers	Illegal practice of the profession (n=3; 1.18%), robberies (n=1; 0.39%)
		8EsocB-I	Insurances-Farmers	High costs (i.e. carcass removal and other insurances) (n=8; 3.14%)

		(n=8; 3.14%)		
		**8EsocB-M (n=50; 19.61%)**	Markets-Farmers	Commercialization problems (n=6; 2.35%), high production costs (n=7; 2.75%), intermediaries (n=3; 1.18%), low demand (n=1; 0.39%), *low profitability (n=28; 10.98%)*, production costs increase (n=2; 0.78%), uncertain profitability (n=6; 2.35%)
		**8EsocB-S (n=81; 31.76%)**	Subsidies-Farmers	Increase subsidy costs (n=1; 0.39%), subsidy dependence (n=5; 1.96%), *subsidy supply distribution inequality (n=18; 7.06%)*, **subsidy supply shortage (n=44; 17.25%),** *subsidy supply uncertainty (n=24; 9.41%)*
**8EsocBpri (n=105; 41.18%)**	Socioeconomic external disturbances-Private infrastructure	**8EsocBpri (n=105; 41.18%)**	Markets-Livestock products	Low demand (n=1; 0.39%), **low profitability (n=98; 38.43%)**, monopoly market (n=1; 0.39%), profitability decrease (n=5; 1.96%), uncertain profitability (n=4; 1.57%)
9IsocB (n=38; 14.90%)	Social internal disturbance-Resource users	*9IsocB (n=38; 14.90%)*	Livestock sector-Farmers	*Demanding job (n=22; 8.63%),* high labor costs (n=2; 0.78%), labor shortage (n=4; 1.57%), weak generational renewal (n=7; 2.75%), skilled labor shortage (n=5; 1.96%)

Table S3. Overview of the response and explanatory variables used in the redundancy analyses (RDA). In the RDA, the vulnerabilities perceived by farmers at the nature level (see Table S2 for a complete list of vulnerabilities) were used as a dichotomous dependent variable (i.e., 1 if the vulnerability was perceived by each farmer or 0 if it was not) and 23 variables used as descriptors of each of the main components of the Coupled Infrastructure System were used as explanatory variables. See the Materials and Methods section for details on statistical analysis, Table S8 for summary statistics and details of the results and Fig. 5 for ordination biplot of the RDA.

Name of variable	Abbreviation	Source	Description
**Response variable**			
Perceived vulnerability		Questionnaires	Vulnerabilities mentioned by farmers and coded according to the Coupled Infrastructure System designed by Anderies et al. (2016). Question: *In your opinion, what are the main problems you face with your farming?* Dummy variable (0/1).
**Explanatory variables**			
(A) Resource			
NDVI	NDVI	Geographical Information System	Average Normalised Difference Vegetation Index (NDVI) between 2001 and 2019, 250 m spatial resolution, derived from MYD13Q1 V6 product (NASA Land Processes Distributed Active Archive Center). Average value was calculated within a 1 km buffer radio around each farmer' farm location. Ln (x+1) transformation was applied to avoid heteroskedasticity.
(B) Resource users			
Age of farmers	Age	Questionnaires	Farmer's age (in years). Ln (x+1) transformation was applied to avoid heteroskedasticity. Question: *Could you please share your age?*
Experience of farmers	Exp	Questionnaires	Farmer's experience as a farmer (in years). Ln (x+1) transformation was applied to avoid heteroskedasticity. Question: *How many years have you been a farmer?*

Male	Male	Questionnaires	When farmer was male. Dummy variable (0/1).
Female	Female	Questionnaires	When farmer was female. Dummy variable (0/1).
Performing transhumance	Transh	Questionnaires	Farmer performs transhumance or transterminance (i.e., short-distance movements). Dummy variable (0/1). Question: *Do you practice transhumance or transterminance with your livestock?*
(Bpri) Private infrastructure			
Number of sheep	Sheep	Questionnaires	Head of sheep per farmer. Ln (x+1) transformation was applied to avoid heteroskedasticity. Question: *How many heads of sheep do you currently have?*
Number of goats	Goats	Questionnaires	Head of goats per farmer. Ln (x+1) transformation was applied to avoid heteroskedasticity. Question: *How many heads of goats do you currently have?*
Number of cattle	Cattle	Questionnaires	Head of cattle per farmer. Ln (x+1) transformation was applied to avoid heteroskedasticity. Question: *How many heads of cattle do you currently have?*
Selling other products	Other_Prod	Questionnaires	Other products sold beyond meat production (e.g., cheese, milk, etc.). Dummy variable (0/1). Question: *Do you produce and sell any products other than meat, such as cheese or milk?*
Head trends	HeadsTrend	Geographical Information System	Changes in the number of livestock heads (i.e., sheep, goats, cattle and pigs) in the last 11 years (2007 – 2018) in the municipality where each farmer' farm was located. Source: MAPA (2019). Trends: (-) Decreasing; (+) Increasing. Ln (x+1) transformation was applied to avoid heteroskedasticity.

Farm trends	FarmsTrend	Geographical Information System	Changes in the number of extensive farms (i.e., sheep, goats, cattle and pigs) in the last 11 years (2007 – 2018) in the municipality where each farmer' farm was located. Source: MAPA (2019). Trends: (-) Decreasing; (+) Increasing. Ln (x+1) transformation was applied to avoid heteroskedasticity.
(C) Public infrastructure providers			
Study area	*CM; PY; CS; CA; NM; FU*	Questionnaires	Farmer' farm located in the Cantabrian Mountains (CM), the Pyrenees (PY), the Central System (CS), Sierras de Cazorla, Segura y Las Villas Natural Park (CA), the Northwest region of Murcia (NM) or Fuerteventura (FU). Nominal variable (CM/PY/CS/CA/NM/FU).
(D) Public infrastructure			
Human footprint	Footprint	Geographical Information System	Human footprint (HF) 2009 (Venter et al. 2016). Average value was calculated within a 1 km buffer radio around each farmer' farm location. HF was downloaded from https://datadryad.org/stash/dataset/doi:10.5061/dryad.052q5. Ln (x+1) transformation was applied to avoid heteroskedasticity.
Protected areas	PA	Geographical Information System	Farmer' farm located in a protected natural area based on MITECO (2019). Dummy variable (0/1).
(E) External disturbances			
(Ebio) Biophysical external disturbances			
Large predators	Pred	Geographical Information System	Presence of large predators (i.e., brown bear and/or wolf) at each farmer' farm location based on the Spanish Inventory on Natural Heritage and Biodiversity (MITECO 2013). Dummy variable (0/1).

Vertebrate richness	VerRich	Geographical Information System	Average number of vertebrate species per 10 x 10 km grids based on the Spanish Inventory on Natural Heritage and Biodiversity (MITECO 2013). Average value was calculated within a 1 km buffer radio around each farmer' farm location. Ln (x+1) transformation was applied to avoid heteroskedasticity.
Temperature	Temp	Geographical Information System	Mean annual temperature (ºC) between 1970 and 2000, with spatial resolution of ~1 km^2, were obtained from WorldClim v2 (Fick and Hijmans 2017). Average value was calculated within a 1 km buffer radio around each farmer' farm location Ln (x+1) transformation was applied to avoid heteroskedasticity.
Temperature seasonality	Temp_sd	Geographical Information System	Annual temperature seasonality, WorldClim v2 (Fick and Hijmans 2017) spatial resolution of ~1 km^2, calculated as the standard deviation (SD) of mean daytime temperature during the year (standard deviation × 100). Average value was calculated within a 1 km buffer radio around each farmer' farm location. Ln (x+1) transformation was applied to avoid heteroskedasticity.
Rainfall	Prec	Geographical Information System	Mean of the total annual rainfall (mm) between 1970 and 2000, was obtained from WorldClim v2 (Fick and Hijmans 2017). Average value was calculated within a 1 km buffer radio around each farmer' farm location. Ln (x+1) transformation was applied to avoid heteroskedasticity.
Rainfall seasonality	Prec_cv	Geographical Information System	Annual rainfall seasonality, WorldClim v2 (Fick and Hijmans 2017) spatial resolution of ~1 km^2 calculated as the coefficient of variation (SD/mean) of the monthly rainfall during the year. Average value was calculated

			within a 1 km buffer radio around each farmer' farm location. Ln (x+1) transformation was applied to avoid heteroskedasticity.
(Esoc) Socioeconomic external disturbances			
Total CAP subsidies	CAP	Geographical Information System	Total amount (in euros) from the European Union's Common Agricultural Policy (CAP) payments at municipality scale based on data provided by the Spanish government (available in https://www.fega.es/es/ficheros_beneficiarios_PAC) which includes beneficiary, the funding line, the municipality and the total amount of money received. We used data from the municipality where each farmer' farm was located. Ln (x+1) transformation was applied to avoid heteroskedasticity.
Pastures CAP subsidies	CAPpasture	Geographical Information System	Index that represents the total amount of subsidies earned in each municipality related to pasture maintenance. This index was created multiplying the total grassland area of the municipality (km^2), the mean payment for grassland maintenance in the region (in euros) and the Coefficient of Admissibility of Pastures (0-100). Average value was calculated within a 1 km buffer radio around each farmer' farm location. Ln (x+1) transformation was applied to avoid heteroskedasticity.

Table S4. Total number of surveys conducted (*Study area*) and summary of explanatory variables obtained from surveys of the farmers for the set of study areas (i.e., total) and in each study area. Percentage or mean (SE) is shown. Descriptions of the variables are provided in Table S3. Further details on location and habitat characteristics of each study area can be obtained through Google Street View by clicking on their names (see Fig. 2 for the location of the study areas).

Variable	Cantabrian Mountains	Pyrenees	Central System	Cazorla	Murcia	Fuerteventura	Total
**(B) Resource users**							
Age (in years)	50.4 (2.2)	49.1 (1.9)	56.6 (2.3)	47.2 (1.2)	53.4 (1.5)	49.3 (1.5)	51.1 (0.7)
Experience (in years)	29.2 (2.8)	33.4 (2.2)	27.1 (2.9)	35.6 (2.2)	32.3 (2.2)	36.2 (2.0)	33.2 (1.0)
Gender							
Male (%)	77.5	84.4	100	100	98.3	86.4	91.0
Female (%)	22.5	15.6	0	0	1.7	13.6	9.0
Performing transhumance (%)	20.0	18.8	0	63.6	0	0	13.7
**(Bpri) Private infrastructure**							
Number of sheep	2.6 (1.2)	470.0 (99.4)	15.2 (12.4)	660.0 (58.8)	635.7 (69.3)	45.3 (13.7)	295.2 (28.0)
Number of goats	18.1 (8.8)	16.7 (6.3)	0.3 (0.3)	29.1 (9.7)	58.8 (16.6)	466.9 (76.3)	131.0 (21.6)
Number of cattle	64.3 (10.4)	39.5 (14.2)	126.3 (15.5)	5.8 (4.0)	0.3 (0.3)	1.3 (0.8)	32.5 (4.2)
Selling other products (%)	0	62.5	0	48.5	41.4	91.5	44.7
(C) Public infrastructure providers							
Study area	40	32	33	33	58	59	255

Table S5. Summary of explanatory variables collected from the geographic information system for all study areas (i.e., total) and by each study area. Percentage or mean (SE) is shown. Descriptions of the variables are provided in Table S3. Further details on location and habitat characteristics of each study area can be obtained through Google Street View by clicking on their names (see Fig. 2 for the location of the study areas).

Variable	Cantabrian Mountains	Pyrenees	Central System	Cazorla	Murcia	Fuerteventura	Total
**(A) Resource**							
NDVI	0.725 (0.05)	0.615 (0.018)	0.492 (0.014)	0.375 (0.012)	0.296 (0.010)	0.119 (0.003)	0.398 (0.013)
**(Bpri) Private infrastructure**							
Number of heads trends	691.8 (5.4)	-7860.7 (747.6)	-583.4 (549.5)	2743.6 (260.5)	-10387.0 (332.0)	-6743.9 (238.0)	-4521.3 (340.0)
Number of farms trends	-21.3 (0.8)	13.9 (2.3)	0.3 (1.4)	135.2 (4.0)	-99.1 (5.6)	-109.9 (5.1)	-32.0 (5.4)
**(D) Public infrastructure**							
Human footprint	7.9 (0.6)	7.7 (0.8)	16.5 (1.6)	3.9 (0.3)	9.3 (0.6)	11.8 (0.6)	9.7 (0.4)
Protected areas (%)	100	15.6	45.5	100	3.4	44.1	47.5
**(Ebio) Biophysical external disturbances**							
Large predators (%)	100	0	69.7	0	0	0	24.7
Vertebrate richness	135.4 (2.7)	119.2 (3.2)	157.9 (5.6)	116.2 (1.5)	89.6 (2.0)	44.0 (0.6)	102 (2.6)
Temperature	9.0 (0.2)	9.2 (0.3)	10.4 (0.2)	9.6 (0.1)	12.5 (0.1)	19.2 (0.1)	12.6 (0.3)
Temperature seasonality	526.2 (4.3)	630.2 (3.6)	656.7 (2.9)	763.6 (1.2)	727.4 (2.0)	271.4 (1.5)	573.7 (11.4)
Rainfall	1160.3 (13.4)	964.6 (29.4)	538.8 (17.8)	626.4 (4.1)	449.7 (6.3)	134.6 (1.7)	587.3 (22.4)

Rainfall seasonality	31.5 (0.2)	18.7 (0.4)	34.9 (0.8)	43.2 (0.1)	40.0 (0.1)	84.1 (0.3)	46.0 (1.4)
**(Esoc) Socioeconomic external disturbances**							
Total CAP subsidies	1386813.6 (66481.7)	992148.8 (98720.3)	1377303.2 (314489.5)	3969254.8 (155804.3)	5605527.0 (436186.0)	2382895.7 (262439.6)	2860271.1 (165476.3)
Pastures CAP subsidies	507138437720.9 (15339729235.9)	451169386485.9 (52457280159.8)	393835640966.3 (76039638934.3)	994177889510.9 (49894544188.3)	259537223331.1 (14330847754.4)	680844589146.7 (25505678195.9)	532354486647.0 (20888939058.8)

Table S6. Number of vulnerabilities identified by surveyed farmers by each study area and for all study areas (i.e., total) at the component and the nature of the vulnerability levels (see Fig. 3 and methods for details on data coding). Values represent mean ± standard deviation and range (x – x) per farmer, and total number of different problems (n).

**Study Area**	**Component level**	**Nature level**
Cantabrian Mountains	2.55 ± 1.54 (0 – 6) (n = 14)	2.63 ± 1.58 (0 – 6) (n = 21)
Pyrenees	2.97 ± 1.28 (1 – 7) (n = 11)	3.22 ± 1.34 (1 – 7) (n = 15)
Central System	1.82 ± 0.88 (0 – 3) (n = 7)	2.00 ± 0.90 (0 – 3) (n = 11)
Cazorla	2.61 ± 1.43 (1 – 6) (n = 13)	2.70 ± 1.42 (1 – 6) (n = 14)
Murcia	2.28 ± 1.02 (0 – 4) (n = 12)	2.38 ± 1.15 (0 – 5) (n = 17)
Fuerteventura	2.49 ± 0.95 (1 – 5) (n = 11)	2.53 ± 0.99 (1 – 5) (n = 15)
Total	*2.44 ± 1.21 (0 – 7) (n = 16)*	*2.55 ± 1.26 (0 – 7) (n = 24)*

Table S7. Complete list of vulnerabilities identified by the surveyed farmers by each study area at the nature of the vulnerability level. Numbers indicate n (%), i.e., number of surveyed farmers (percentage relative to the total of farmers by each study area) who identified each vulnerability. Study area code: Cantabrian Mountains (CM; n=40); Pyrenees (PY; n=32); Central System (CS; n=33); Sierras de Cazorla, Segura y Las Villas Natural Park (CA; n=33); Northwest region of Murcia (NM; n=58); Fuerteventura (FU; n=59). Values 5 - 15% are in italic and >15% in bold. See Table S2 for vulnerabilities for all study areas.

Name	Description	Coding examples	CM (%)	PY (%)	CS (%)	CA (%)	NM (%)	FU (%)
1AB	Pastures-Farmers		*4 (10.00)*	0	0	*3 (9.09)*	*4 (6.90)*	2 (3.39)
		Excess livestock	2 (5.00)	0	0	0	0	0
		Pasture shortage	*4 (10.00)*	0	0	*3 (9.09)*	*4 (6.90)*	2 (3.39)
1AextB	Food input-Farmers		0	0	0	0	0	**9 (15.25)**
		Cereal shortage	0	0	0	0	0	1 (1.69)
		Difficulties in growing pastures	0	0	0	0	0	1 (1.69)
		Food importation	0	0	0	0	0	*7 (11.86)*
		High costs	0	0	0	0	0	2 (3.39)
		Low quality of animal food	0	0	0	0	0	2 (3.39)
BBpri	Farmers-Farms		1 (2.50)	*3 (9.38)*	0	1 (3.03)	*4 (6.90)*	1 (1.69)
		High investment	0	0	0	0	1 (1.72)	0
		Livestock diseases	1 (2.50)	*3 (9.38)*	0	1 (3.03)	2 (3.45)	1 (1.69)
		Livestock dung acumulation	0	0	0	0	1 (1.72)	0
		Livestock losses	0	0	0	0	1 (1.72)	0
2CB	Government-Farmers		*3 (7.50)*	**5 (15.63)**	*2 (6.06)*	*2 (6.06)*	0	*5 (8.47)*
		Cheats and lack of trust in wildlife attacks	1 (2.50)	0	0	0	0	0
		Corruption and lack of information	0	0	0	0	0	1 (1.69)

		Ecologists	0	0	1 (3.03)	0	0	0
		Government´s lack of support to the sector	2 (5.00)	0	0	1 (3.03)	0	*4 (6.78)*
		Government´s lack of support to young farmers	0	0	0	1 (3.03)	0	0
		Low cooperation among farmers	0	0	1 (3.03)	0	0	0
5Dsoc(AB)	Institutions-(Farmers-Pasture management)		*5 (12.50)*	1 (3.13)	0	0	1 (1.72)	0
		Economic resources shortage	1 (2.50)	0	0	0	0	0
		Limits and management	1 (2.50)	0	0	0	0	0
		Mountain and agricultural land management	0	1 (3.13)	0	0	0	0
		Not allowed to manage shrub colonization	2 (5.00)	0	0	0	0	0
		Pasture shortage	2 (5.00)	0	0	0	1 (1.72)	0
6DphyB	Roads-Farmers		*4 (10.00)*	0	0	*2 (6.06)*	0	0
		Lack of roads	2 (5.00)	0	0	0	0	0
		Not allowed to create roads	2 (5.00)	0	0	0	0	0
		Poor conditions of roads	1 (2.50)	0	0	*2 (6.06)*	0	0
6DsocB	Institutions-Farmers		**11 (27.50)**	**15 (46.88)**	**5 (15.15)**	**5 (15.15)**	*6 (10.34)*	*8 (13.56)*
		Bureaucracy	*4 (10.00)*	**12 (37.50)**	0	*3 (9.09)*	1 (1.72)	1 (1.69)
		Cheats in number of livestock	0	0	0	0	0	2 (3.39)
		Lack of electric supply	0	0	0	0	0	1 (1.69)
		Lack of regulations	0	0	1 (3.03)	0	0	0
		Legal requirements for carcass disposal	0	1 (3.13)	0	0	1 (1.72)	0

		Legal requirements for livestock numbers	0	0	0	0	0	1 (1.69)
		Legal requirements for livestock sanitation	0	0	0	0	1 (1.72)	0
		Livestock sanitation poorly done	*3 (7.50)*	0	0	0	0	0
		Low monetary compensations	1 (2.50)	0	0	0	0	0
		Mandatory carcass removal insurance	0	*2 (6.25)*	0	0	2 (3.45)	*3 (5.08)*
		Many inspections	0	0	0	1 (3.03)	0	0
		Many legal requirements	0	0	0	1 (3.03)	1 (1.72)	1 (1.69)
		More carcass removal options	0	1 (3.13)	0	0	0	0
		Restrictions to animal movements	0	0	1 (3.03)	0	0	0
		Restrictions to number of animals	1 (2.50)	0	0	0	0	0
		Sanitation	1 (2.50)	1 (3.13)	*3 (9.09)*	0	0	0
		Sanitation costs	0	0	0	1 (3.03)	2 (3.45)	0
		Uncertain compensations	1 (2.50)	0	0	0	0	0
		Very rigorous livestock sanitation	2 (5.00)	0	0	0	0	0
7EbioA	Climate-Pasture, Wildlife-Pasture		**10 (25.00)**	*3 (9.38)*	0	**5 (15.15)**	**10 (17.24)**	**15 (25.42)**
7EbioA-C	Climate-Pasture		1 (2.50)	1 (3.13)	0	**5 (15.15)**	**9 (15.52)**	**15 (25.42)**
		Cereal shortage	0	0	0	0	1 (1.72)	0
		Difficulties in growing pastures	0	0	0	0	0	1 (1.69)
		Drought	0	0	0	1 (3.03)	1 (1.72)	**9 (15.25)**
		Inclement weather	0	1 (3.13)	0	0	0	0

		Pasture shortage	0	1 (3.13)	0	0	2 (3.45)	2 (3.39)
		Rainfall shortage	0	0	0	*4 (12.12)*	*8 (13.79)*	*5 (8.47)*
		Supplementary feeding needed	0	0	0	0	1 (1.72)	0
		Water shortage	1 (2.50)	0	0	0	0	1 (1.69)
7EbioA-W	Wildlife-Pasture		**9 (22.50)**	*2 (6.25)*	0	0	1 (1.72)	0
		Grazing competence with ungulates	*3 (7.50)*	0	0	0	0	0
		Wild boar crop damage	0	0	0	0	1 (1.72)	0
		Wild boar rooting	**9 (22.50)**	*2 (6.25)*	0	0	0	0
7EbioB	Climate-Farmers		*3 (7.50)*	0	0	1 (3.03)	*8 (13.79)*	0
		Inclement weather	*3 (7.50)*	0	0	1 (3.03)	*8 (13.79)*	0
		Isolated people	1 (2.50)	0	0	0	0	0
7EbioBpri	Wildlife-Livestock		**28 (70.00)**	**5 (15.63)**	**12 (36.36)**	1 (3.03)	1 (1.72)	0
		Disease transmission by ungulates	*3 (7.50)*	0	0	0	0	0
		Increase of wolves	1 (2.50)	0	0	0	0	0
		Wildlife attacks	**27 (67.50)**	**5 (15.63)**	**12 (36.36)**	1 (3.03)	1 (1.72)	0
7EbioDphy	Natural vegetation-Roads		1 (2.50)	0	0	0	0	0
		Colonization of roads by shrubs	1 (2.50)	0	0	0	0	0
8EsocA	Markets-Pastures, Markets-Water		0 (0)	1 (3.13)	0 (0)	**7 (21.21)**	*8 (13.79)*	2 (3.39)
8EsocA-P	Markets-Pastures		0	1 (3.13)	0	**7 (21.21)**	*8 (13.79)*	0
		Cannot improve facilities	0	0	0	0	1 (1.72)	0
		High costs	0	1 (3.13)	0	**7 (21.21)**	*8 (13.79)*	0
8EsocA-W	Markets-Water		0	0	0	0	0	2 (3.39)

		Difficulties in growing pastures	0	0	0	0	0	2 (3.39)
		High costs	0	0	0	0	0	2 (3.39)
8EsocAext	Markets-External input, Markets-Food input		*4 (10.00)*	**16 (50.00)**	*3 (9.09)*	**12 (36.36)**	**32 (55.17)**	**46 (77.97)**
8EsocAext-ExtI	Markets-External input		1 (2.50)	*4 (12.50)*	0	0	*4 (6.90)*	0
		Cost increase	1 (2.50)	1 (3.13)	0	0	0	0
		High costs	0	*3 (9.38)*	0	0	*4 (6.90)*	0
8EsocAext-ExtF	Markets-Food input		*4 (10.00)*	**16 (50.00)**	*3 (9.09)*	**12 (36.36)**	**32 (55.17)**	**46 (77.97)**
		Cost increase	2 (5.00)	*3 (9.38)*	0	0	2 (3.45)	0
		High costs	2 (5.00)	**13 (40.63)**	*3 (9.09)*	**12 (36.36)**	**30 (51.72)**	**46 (77.97)**
		Prices uncertainty	0	0	0	0	1 (1.72)	0
8EsocB	Credits & Taxes-Farmers, Economic crisis-Farmers, Free riding-Farmers, Insurances-Farmers, Markets-Farmers, Subsidies-Farmers		**16 (40.00)**	**19 (59.38)**	**24 (72.73)**	**19 (57.58)**	**21 (36.21)**	**34 (57.63)**
8EsocB-C	Credits & Taxes-Farmers		1 (2.50)	0	*4 (12.12)*	0	0	0
		Cost increase	0	0	1 (3.03)	0	0	0
		Credit shortage	0	0	1 (3.03)	0	0	0
		High costs	0	0	*2 (6.06)*	0	0	0
		High interests	0	0	1 (3.03)	0	0	0
		Many taxes	1 (2.50)	0	0	0	0	0
8EsocB-E	Economic crisis-Farmers		1 (2.50)	0	1 (3.03)	0	0	2 (3.39)
		Economic crisis	1 (2.50)	0	1 (3.03)	0	0	2 (3.39)
8EsocB-Fr	Free riding-Farmers		1 (2.50)	0	1 (3.03)	0	1 (1.72)	1 (1.69)

		Illegal practice of the profession	1 (2.50)	0	1 (3.03)	0	0	1 (1.69)
		Robberies	0	0	0	0	1 (1.72)	0
8EsocB-I	Insurances-Farmers		1 (2.50)	*4 (12.50)*	0	0	1 (1.72)	2 (3.39)
		High costs	1 (2.50)	*4 (12.50)*	0	0	1 (1.72)	2 (3.39)
8EsocB-M	Markets-Farmers		*5 (12.50)*	**5 (15.63)**	**13 (39.39)**	**6 (18.18)**	**16 (27.59)**	*5 (8.47)*
		Commercialization problems	2 (5.00)	1 (3.13)	0	0	1 (1.72)	2 (3.39)
		High production costs	1 (2.50)	0	*3 (9.09)*	1 (3.03)	2 (3.45)	0
		Intermediaries	0	1 (3.13)	0	0	0	2 (3.39)
		Low demand	0	0	1 (3.03)	0	0	0
		Low profitability	2 (5.00)	*2 (6.25)*	**9 (27.27)**	*3 (9.09)*	**12 (20.69)**	0
		Production costs increase	0	1 (3.13)	1 (3.03)	0	0	0
		Uncertain profitability	0	0	1 (3.03)	*2 (6.06)*	2 (3.45)	1 (1.69)
8EsocB-S	Subsidies-Farmers		**9 (22.50)**	**14 (43.75)**	**11 (33.33)**	**16 (48.48)**	*5 (8.62)*	**26 (44.07)**
		Increase subsidy costs	0	0	0	0	1 (1.72)	0
		Subsidy dependence	0	*3 (9.38)*	0	1 (3.03)	0	1 (1.69)
		Subsidy supply distribution inequality	0	**10 (31.25)**	0	1 (3.03)	1 (1.72)	*6 (10.17)*
		Subsidy supply shortage	**9 (22.50)**	*3 (9.38)*	**11 (33.33)**	**5 (15.15)**	*3 (5.17)*	**13 (22.03)**
		Subsidy supply uncertainty	1 (2.50)	1 (3.13)	0	**10 (30.30)**	1 (1.72)	**11 (18.64)**
8EsocBpri	Markets-Livestock products		**10 (25.00)**	**15 (46.88)**	**12 (36.36)**	**22 (66.67)**	**22 (37.93)**	**24 (40.68)**
		Low demand	1 (2.50)	0	0	0	0	0
		Low profitability	**8 (20.00)**	**15 (46.88)**	**11 (33.33)**	**20 (60.61)**	**20 (34.48)**	**24 (40.68)**
		Monopoly market	0	0	0	0	1 (1.72)	0

		Profitability decrease	2 (5.00)	1 (3.13)	1 (3.03)	0	1 (1.72)	0
		Uncertain profitability	0	0	0	*3 (9.09)*	1 (1.72)	0
9IsocB	Livestock sector-Farmers		2 (5.00)	**12 (37.50)**	*2 (6.06)*	**6 (18.18)**	**15 (25.86)**	1 (1.69)
		Demanding job	2 (5.00)	*3 (9.38)*	0	*4 (12.12)*	**12 (20.69)**	1 (1.69)
		High labor costs	0	0	0	0	2 (3.45)	0
		Labor shortage	0	*3 (9.38)*	1 (3.03)	0	0	0
		Weak generational renewal	0	**5 (15.63)**	0	*2 (6.06)*	0	0
		Skilled labor shortage	0	*2 (6.25)*	1 (3.03)	0	2 (3.45)	0

Table S8. Summary statistics and results of the four different redundancy analyses (RDA) conducted showing the relationship between the vulnerabilities identified by farmers (see gray points in Fig. 5 for graphical representation and Table S2 for complete list of vulnerabilities) and the explanatory variables describing the components of the Coupled Infrastructure System (see the Materials and Methods section and Table S3 for descriptions of the explanatory variables). Explanatory variables were related to the non-human components (A resources, and D public infrastructure), the human components (B resource users and C public infrastructure providers), the private infrastructure of the resource users (Bpri), the biophysical external disturbances (Ebio) and the socioeconomic external disturbances (Esoc) of the Coupled Infrastructure System. See the Materials and Methods section for details on statistical analysis and Fig. 5 for ordination biplot of the RDA. Significance codes: *** $p \leq 0.001$; ** $p \leq 0.01$; * $p \leq 0.05$; n.s. $P \geq 0.05$.

	Eigenvalue	Variance (%)	Significance
(A) Resource; (D) Public infrastructure			
RDA 1	0.113	5.890	**
RDA 2	0.018	0.940	*
Protected areas (*PA*)	-	0.018	**
NDVI	-	0.032	***
Human footprint (*Footprint*)	-	0.017	**
(B) Resource users; (C) Public infrastructure providers			
RDA 1	0.192	9.970	*
RDA 2	0.109	5.690	n.s.
Study area (*CM; PY; CS; CA; NM; FU*)	-	0.134	***
Age of farmers (*Age*)	-	0.014	*
Experience of farmers (*Exp*)	-	0.479	*
Male	-	0	n.s.
Female	-	0	n.s.
Performing transhumance (*Transh*)	-	0.006	n.s.
(Bpri) Private infrastructure			
RDA 1	0.271	14.100	*
RDA 2	0.144	7.470	n.s.
Number of sheep (*Sheep*)	-	0.471	n.s.
Number of goats (*Goats*)	-	0.390	n.s.
Number of cattle (*Cattle*)	-	0.010	n.s.
Selling other products (*Other_Prod*)	-	0.008	n.s.
Number of heads trends (*HeadsTrend*)	-	0.010	n.s.
Number of farms trends (*FarmsTrend*)	-	0.014	*
(E) External disturbances			
RDA 1	0.139	7.220	**
RDA 2	0.040	2.060	**
(Ebio) Biophysical external disturbances			
Large predators (*Pred*)		0.028	***
Vertebrate richness (*VerRich*)		0.007	n.s.
Temperature (*Temp*)		0.017	**
Temperature seasonality (*Temp_sd*)		0.013	*
Rainfall (*Prec*)		0.021	***
Rainfall seasonality (*Prec_cv*)		0.005	n.s.
(Esoc) Socioeconomic external disturbances			
Total CAP subsidies (*CAP*)		0.006	n.s.
Pastures CAP subsidies (CAPpasture)		0.011	n.s.

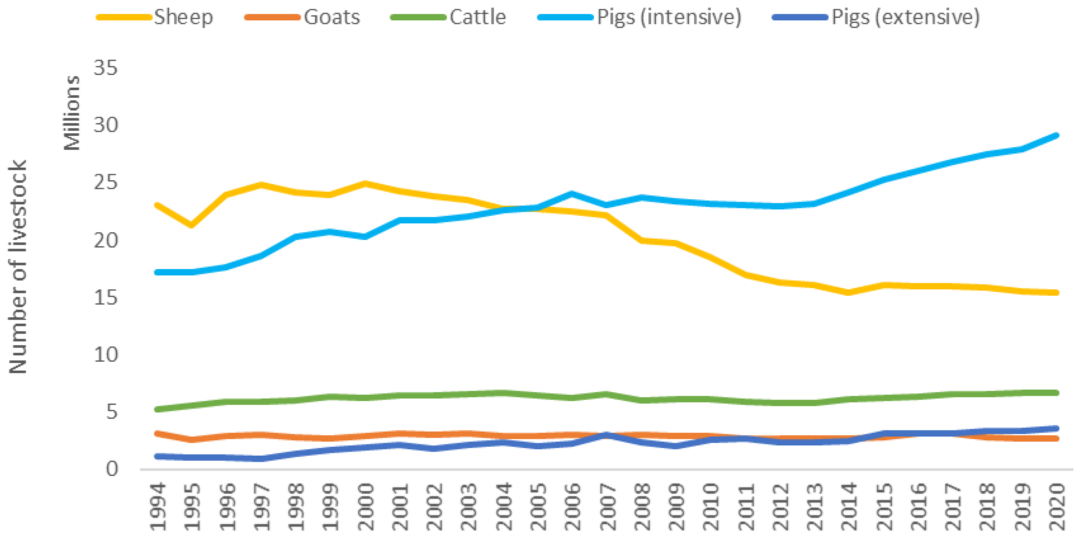

Fig. S1. Evolution of the number of livestock (sheep, goats, cattle, and pigs) in the last three decades (1994 - 2020) in Spain. Source: MAPA (2019).

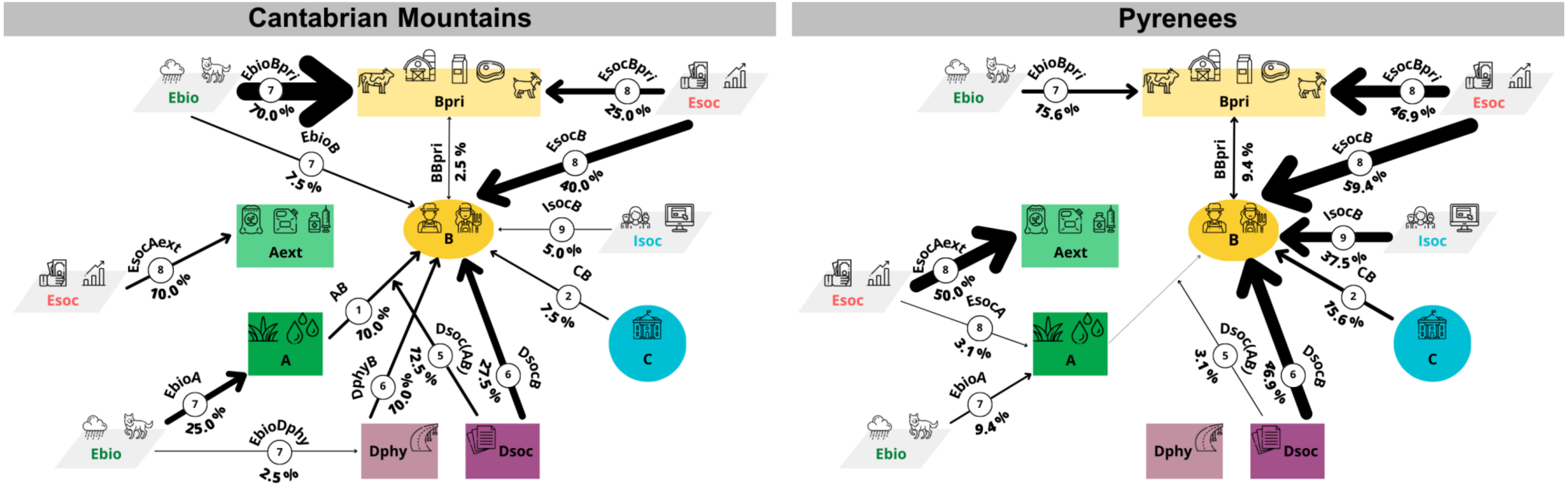

Fig. S2. Representation of the vulnerabilities identified by the surveyed farmers in the Cantabrian Mountains and the Pyrenees. The thickness of the arrows and the numbers next to the arrows indicate the degree to which each vulnerability mentioned by farmers is likely to affect the sustainability or maintenance of livestock farming systems, based on the percentage of farmers who mentioned each vulnerability (i.e., each link), whereas the links not mentioned by farmers are not shown. Components: Resource (A); External resource (Aext); Resource users (B); Private infrastructure (Bpri); Public infrastructure providers (C); Social public infrastructure (Dsoc), Physical public infrastructure (Dphy); Biophysical external disturbances (Ebio); Socioeconomic external disturbances (Esoc); Social internal disturbances (Isoc). Coding examples of each vulnerability are listed in Table S7. Total number surveyed farmers by each study area (see Fig. 2 for the location of the study areas): Cantabrian Mountains (n=40); Pyrenees (n=32).

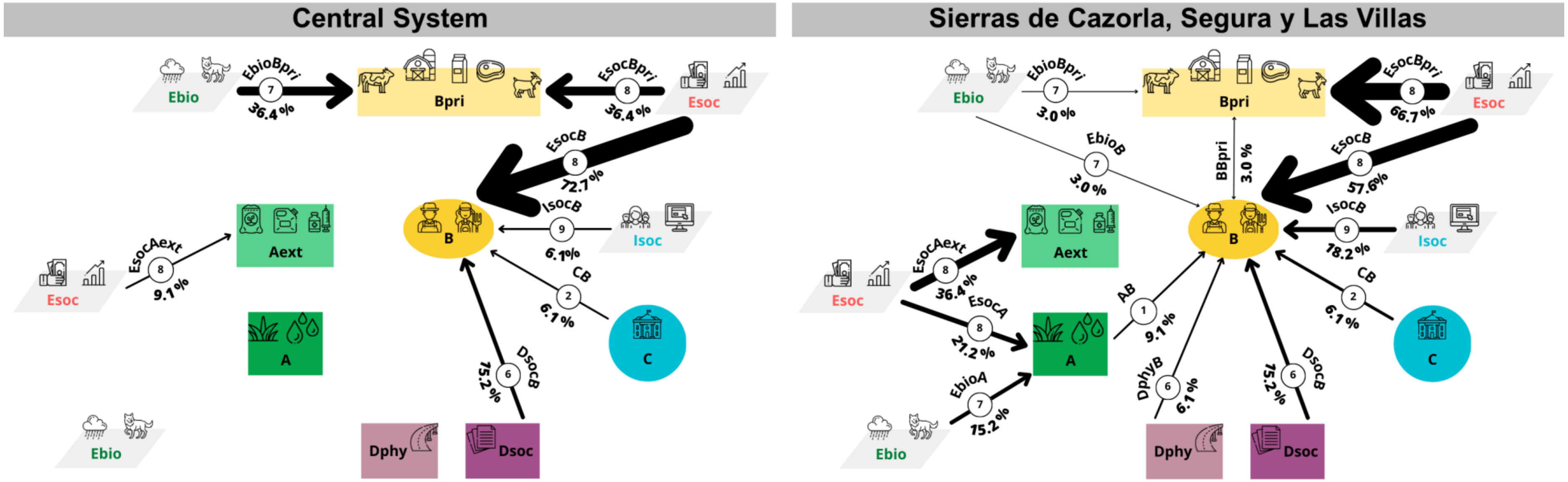

Fig. S3. Representation of the vulnerabilities identified by the surveyed farmers in the Central System and Sierras de Cazorla, Segura y Las Villas Natural Park. The thickness of the arrows and the numbers next to the arrows indicate the degree to which each vulnerability mentioned by farmers is likely to affect the sustainability or maintenance of livestock farming systems, based on the percentage of farmers who mentioned each vulnerability (i.e., each link), whereas the links not mentioned by farmers are not shown. Components: Resource (A); External resource (Aext); Resource users (B); Private infrastructure (Bpri); Public infrastructure providers (C); Social public infrastructure (Dsoc), Physical public infrastructure (Dphy); Biophysical external disturbances (Ebio); Socioeconomic external disturbances (Esoc); Social internal disturbances (Isoc). Coding examples of each vulnerability are listed in Table S7. Total number surveyed farmers by each study area (see Fig. 2 for the location of the study areas): Central System (n=33); Sierras de Cazorla, Segura y Las Villas Natural Park (n=33).

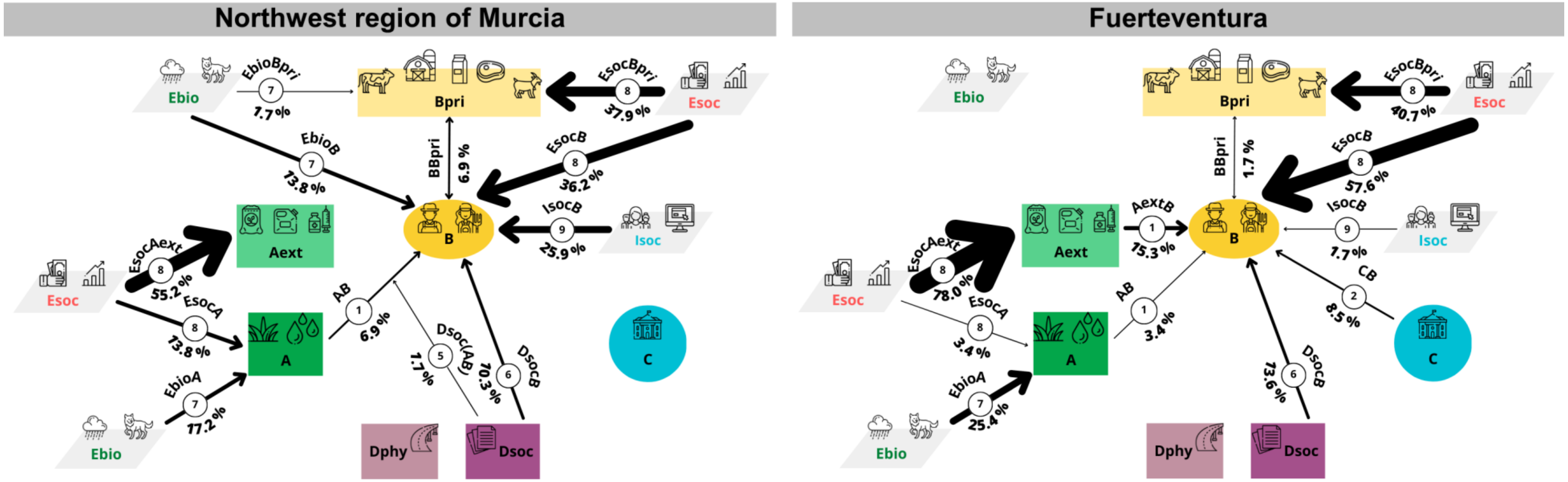

Fig. S4. Representation of the vulnerabilities identified by the surveyed farmers in the northwest region of Murcia and Fuerteventura. The thickness of the arrows and the numbers next to the arrows indicate the degree to which each vulnerability mentioned by farmers is likely to affect the sustainability or maintenance of livestock farming systems, based on the percentage of farmers who mentioned each vulnerability (i.e., each link), whereas the links not mentioned by farmers are not shown. Components: Resource (A); External resource (Aext); Resource users (B); Private infrastructure (Bpri); Public infrastructure providers (C); Social public infrastructure (Dsoc), Physical public infrastructure (Dphy); Biophysical external disturbances (Ebio); Socioeconomic external disturbances (Esoc); Social internal disturbances (Isoc). Coding examples of each vulnerability are listed in Table S7. Total number surveyed farmers by each study area (see Fig. 2 for the location of the study areas): Northwest region of Murcia (n=58); Fuerteventura (n=59).

Supporting references

Anadón, J. D., A. Giménez, R. Ballestar, and I. Pérez. 2009. Evaluation of local ecological knowledge as a method for collecting extensive data on animal abundance. *Conservation Biology* 23: 617–625. doi:10.1111/j.1523-1739.2008.01145.x

Anderies, J. M., M. A. Janssen, and E. Ostrom. 2004. A framework to analyze the robustness of social-ecological systems from an institutional perspective. *Ecology and Society* 9: 18. doi:10.5751/es-00610-090118.

Anderies, J. M., M. A. Janssen, and E. Schlager. 2016. Institutions and the performance of coupled infrastructure systems. *International Journal of the Commons* 10: 495–516. doi:10.18352/ijc.651.

Bartlett, J. E., J. W. Kotrlik, and C. C. Higgins. 2001. Organizational research: Determining appropriate sample size in survey research. *Information Technology, Learning, and Performance Journal* 19: 43–50.

Cochran, W. G. 1977. *Sampling techniques*. New York: John Wiley & Sons.

Fick, S. E., and R. J. Hijmans. 2017. WorldClim 2: New 1km spatial resolution climate surfaces for global land areas. *International Journal of Climatology* 37(12): 4302–4315. doi:10.1002/joc.5086.

Heitz, C., S. Spaeter, A.-V. Auzet, and S. Glatron. 2009. Local stakeholders' perception of muddy flood risk and implications for management approaches: A case study in Alsace (France). *Land Use Policy* 26: 443–451. doi:10.1016/j.landusepol.2008.05.008

La Rosa, D., and R. Privitera. 2013. Characterization of non-urbanized areas for land-use planning of agricultural and green infrastructure in urban contexts. *Landscape and Urban Planning* 109: 94–106. doi:10.1016/j.landurbplan.2012.05.012

MAPA. 2019. *Spanish Ministry of Agriculture, Fisheries and Food.* Available at: https://www.mapa.gob.es.

Martín-López, B., M. García-Llorente, I. Palomo, and C. Montes. 2011. The conservation against development paradigm in protected areas: Valuation of ecosystem services in the Doñana social–ecological system (southwestern Spain). *Ecological Economics* 70: 1481–1491. doi:10.1016/j.ecolecon.2011.03.009

Mekasha, A., B. Gerard, K. Tesfaye, L. Nigatu, and A. J. Duncan. 2014. Inter-connection between land use/land cover change and herders'/farmers' livestock feed resource management strategies: A case study from three Ethiopian eco-environments. *Agriculture, Ecosystems & Environment* 188: 150–162. doi:10.1016/j.agee.2014.02.022

MITECO. 2013. *Spanish Inventory of Natural Heritage and Biodiversity.* Available at: https://www.miteco.gob.es.

MITECO. 2019. *Protected Natural Areas in Spain.* Available at: https://www.miteco.gob.es.

Morales-Reyes, Z., B. Martín-López, M. Moleón, P. Mateo-Tomás, F. Botella, A. Margalida, J. A. Donázar, G. Blanco, et al. 2018. Farmer Perceptions of the Ecosystem Services Provided by Scavengers: What, Who, and to Whom. *Conservation Letters* 11: e12392. doi:10.1111/conl.12392

Newing, H. 2010. *Conducting research in conservation: Social science methods and practice*. New York: Taylor & Francis.

Oksanen, J., F. G. Blanchet, M. Friendly, R. Kindt, P. Legendre, D. McGlinn, P. R. Minchin, R. B. O'Hara, et al. 2019. vegan: Community Ecology Package. R package version 2.5-6. https://CRAN.R-project.org/package=vegan.

Pérez, I., A. Giménez, and A. Pedreño. 2012. Impacts of exurban sprawl: The effects of the perceptions and practices of new residents toward the spur-thighed tortoise (*Testudo graeca*). *Wildlife Society Bulletin* 36: 531–537. doi: 10.1002/wsb.162

R Core Team. 2020. R: A language and environment for statistical computing. R Foundation for Statistical Computing, Vienna, Austria. Available at: http://www.R-project.org.

Venter, O., E. W. Sanderson, A. Magrach, J. R. Allan, J. Beher, K. R. Jones, H. P. Possingham, W. F. Laurance, et al. 2016. Sixteen years of change in the global terrestrial human footprint and implications for biodiversity conservation. Nature Communications 7: 12558. doi:10.1038/ncomms12558.
